# Risk factors for recurrent laryngeal nerve injury following thyroid surgery: a systematic review and meta-analysis

**DOI:** 10.3389/fsurg.2025.1731701

**Published:** 2026-01-07

**Authors:** Xiang Yang, Wuzhou Ouyang, Peng Ma

**Affiliations:** 1 Beijing Anzhen Nanchong Hospital of Capital Medical University & Nanchong Central Hospital, Nanchong, China; 2 Sichuan Nanchong No. 1 Middle School, Nanchong, China

**Keywords:** meta-analysis, recurrent laryngeal nerve injury, risk factors, systematic review, thyroid surgery

## Abstract

**Background:**

Recurrent laryngeal nerve injury is one of the most common and severe complications in thyroid surgery, potentially leading to postoperative hoarseness, dysphagia, or even airway obstruction. Although numerous studies have investigated its risk factors, findings remain inconsistent. This systematic review and meta-analysis aim to synthesize existing evidence and explore risk factors for recurrent laryngeal nerve injury following thyroid surgery.

**Methods:**

A systematic search was conducted in PubMed, Embase, Web of Science, and the Cochrane Library databases from their inception to October 1, 2025, to identify observational studies and randomized controlled trials investigating risk factors for recurrent laryngeal nerve injury following thyroid surgery. Two researchers independently performed literature screening, data extraction, and quality assessment using the Newcastle–Ottawa Scale (NOS). Stata 15 software was used to calculate pooled odds ratios (OR) and 95% confidence intervals (CI).

**Results:**

A total of 20 articles(*N* = 108,343) included, meta-analysis results suggest that older age [OR = 1.45, 95% CI (1.26, 1.66)], Female [OR = 1.15, 95% CI (1.03, 1.28)], extended thyroidectomy [OR = 1.65, 95% CI (1.20, 2.27)], node dissection [OR = 2.28, 95% CI (1.67, 3.09)],reoperation [OR = 2.16, 95% CI (1.86, 2.50)], retrosternal goitre [OR = 2.85, 95% CI (1.87, 4.35)], lack of neuromonitoring [OR = 1.64, 95% CI (1.31, 2.06)] may be associated with RLNI following thyroid surgery.

**Conclusion:**

This study indicates that older age, female gender, extended thyroidectomy, lymph node dissection, reoperation, retrosternal goiter, and absence of nerve monitoring mya be independent risk factors for recurrent laryngeal nerve injury following thyroid surgery.

**Systematic Review Registration:**

https://www.crd.york.ac.uk/PROSPERO/view/CRD420251106124, identifier CRD420251106124.

## Background

Thyroid surgery is one of the primary methods for treating benign and malignant thyroid diseases, including thyroid nodules, hyperthyroidism, and thyroid cancer (1). With continuous advancements in ultrasound technology, anesthesia management, and microsurgical techniques, the safety of thyroid surgery has significantly improved (2). However, recurrent laryngeal nerve injury (RLNI) remains one of the most common and clinically significant complications in thyroid surgery. The recurrent laryngeal nerve controls the movement of most intrinsic laryngeal muscles, serving as a vital structure for maintaining vocal cord function and respiratory capacity (3). Its injury can cause symptoms such as hoarseness, dysphonia, dysphagia, and coughing. In severe cases, respiratory distress may occur, necessitating tracheotomy to preserve airway patency (4). This complication not only impacts patients' quality of life and social interactions but also imposes long-term effects on postoperative recovery and mental health (5). Recurrent laryngeal nerve injury is typically classified as temporary or permanent. Literature reports indicate that the incidence of temporary RLNI ranges from approximately 1% to 10%, while permanent injury occurs in about 0.5% to 2% of cases (6). However, in patients with malignant thyroid tumors, giant goiters, or those undergoing repeat surgery, this proportion can reach 5% or higher (7). The mechanism of RLNI is complex, involving not only direct transection but also intraoperative traction, thermal injury, compression, or compromised neural blood supply. Additionally, anatomical variations of the recurrent laryngeal nerve constitute another significant cause of injury (8). Some patients exhibit atypical nerve courses or branch variations, particularly in the right lower pole region or in cases of non-recurrent laryngeal nerves. Failure to accurately identify these structures during surgery can easily lead to inadvertent injury (9, 10).

To reduce RLNI incidence, various protective measures have been proposed in recent years, including the application of intraoperative nerve monitoring, precise microsurgical dissection, and accumulating surgeon experience (11). Despite technological advancements, RLNI remains unavoidable. Some studies suggest that reoperation, malignant lesions, and central zone dissection significantly increase the risk of nerve injury, while others find no significant correlation (12, 13). Limited sample sizes in individual studies, substantial differences in study designs, and inadequate control of confounding factors restrict the reliability and consistency of results (14). Against this backdrop, it is necessary to systematically integrate and quantitatively analyze existing literature through systematic reviews and meta-analyses to identify independent risk factors for recurrent laryngeal nerve injury after thyroid surgery. Systematic reviews and meta-analyses can synthesize findings from multiple studies, reduce random error, enhance statistical power, and provide higher-quality evidence-based guidance for clinical practice. By identifying high-risk factors, clinicians can thoroughly assess patient risk preoperatively and develop individualized surgical strategies. During surgery, emphasis on nerve anatomy identification and protection, supplemented by nerve monitoring technology, when necessary, can effectively reduce the incidence of recurrent laryngeal nerve injury, thereby improving surgical safety and patient outcomes.

## Methods

This systematic evaluation and meta-analysis will strictly follow the PRISMA (Preferred Reporting Items for Systematic Reviews and Meta-Analyses) guidelines (15). And it is registered in Prospero with registration number CRD420251106124.

## Literature retrieval

A systematic search was conducted in PubMed, Embase, Web of Science, and the Cochrane Library databases from their inception to October 1, 2025, to identify literature related to risk factors for recurrent laryngeal nerve injury following thyroid surgery. The search strategy combined Medical Subject Headings (MeSH) terms and free-text keywords, with primary search terms including: “thyroid surgery,” “thyroidectomy,” “recurrent laryngeal nerve injury,” “nerve damage,” “risk factor,” “predictor,” and “complication”. The detailed search strategy is provided in Supplementary Table S1. To minimize omissions, reference lists of included studies and citations from relevant reviews were manually searched.

## Literature inclusion and exclusion criteria

### Inclusion criteria

Study Type: Prospective or retrospective observational studies (cohort studies, case-control studies, cross-sectional studies) and randomized controlled trials.
Study Population: Patients undergoing any type of thyroid surgery.
Outcome Measures: Clearly reported incidence of recurrent laryngeal nerve injury (including temporary or permanent).
Study Results: Provided or calculable effect sizes for risk factors (OR values, RR values, and their 95% confidence intervals).
Assessable literature quality with complete data.

### Exclusion criteria

Studies with duplicate publications or overlapping data.
Case reports, conference abstracts, reviews, commentaries, or animal studies.
Studies failing to clearly distinguish types of recurrent laryngeal nerve injury or lacking extractable data.
Studies where full-text access is unavailable.

### Study selection

During the literature screening process, two researchers independently used EndNote 21 software to initially screen the literature obtained from the search, first through the titles and abstracts, and then to exclude literature that clearly did not meet the inclusion criteria. Subsequently, the remaining literature was reviewed by reading the full text in its entirety to further determine whether it met the inclusion and exclusion criteria. In case of disagreement between the two researchers during the screening process, it would be resolved through discussion and negotiation; if the negotiation still failed to reach a consensus, a third researcher would be invited to adjudicate to ensure the objectivity and consistency of the screening process.

### Data extractions

This study was conducted by two researchers who independently extracted relevant data from the eligible literature using an Excel sheet based on the inclusion criteria. The extraction included the basic information of the study (first author, year of publication, country and study design), the basic characteristics of the study population (sample size, number of infections, type of infections, gender, and mean age), the statistical model used in the regression analysis, and diagnosis of cognitive impairment. In the process of data extraction, if two investigators disagreed on the data, it would be resolved through negotiation, and if no agreement could be reached, a third investigator would adjudicate to ensure the accuracy and consistency of data extraction.

### Quality evaluation

The types of studies included in this study will be assessed using different quality assessment tools: for case-control and cohort studies, the NOS (Newcastle-Ottawa Scale) (16) quality assessment tool will be used, which evaluates the intrinsic bias of the studies through three main domains (study selectivity, comparability, and assessment of outcomes), focusing on sample selection, the relationship between exposure and relationship between exposure and outcome, and control of confounders; these quality assessment tools ensure that the included studies have a high-quality evidence base.

### Statistical analysis

In this study, the risk ratio (OR) and the corresponding 95% confidence interval (CI) of each included study were combined using Stata 15 software. First, for each study, we extracted the corresponding effect size OR and its 95% confidence interval. To combine these ORs, we pooled them using a random effects model, which can account for heterogeneity between studies, variability in effect sizes across studies. ORs and 95% CIs were calculated for each study and combined into an overall effect size. Heterogeneity of the model was assessed by the I² statistic; if the I² was greater than 50%, it was considered that there was a high degree of heterogeneity and that the sources of heterogeneity needed to be further explored. For high heterogeneity, we may conduct sensitivity analyses to identify potential factors that may affect the combined effect sizes. Asymmetry in the funnel plot indicates a higher likelihood of publication bias, which will be further evaluated using Egger's test. *P* value < 0.05 suggests the presence of publication bias, while a *P* value > 0.05 suggests otherwise. If necessary, the trim-and-fill method will be used for further confirmation. The combined effect sizes will be reported as ORs and their 95% CIs to allow for interpretation of results and statistical inference.

## Results

### Literature screening results

**Figure 1:**
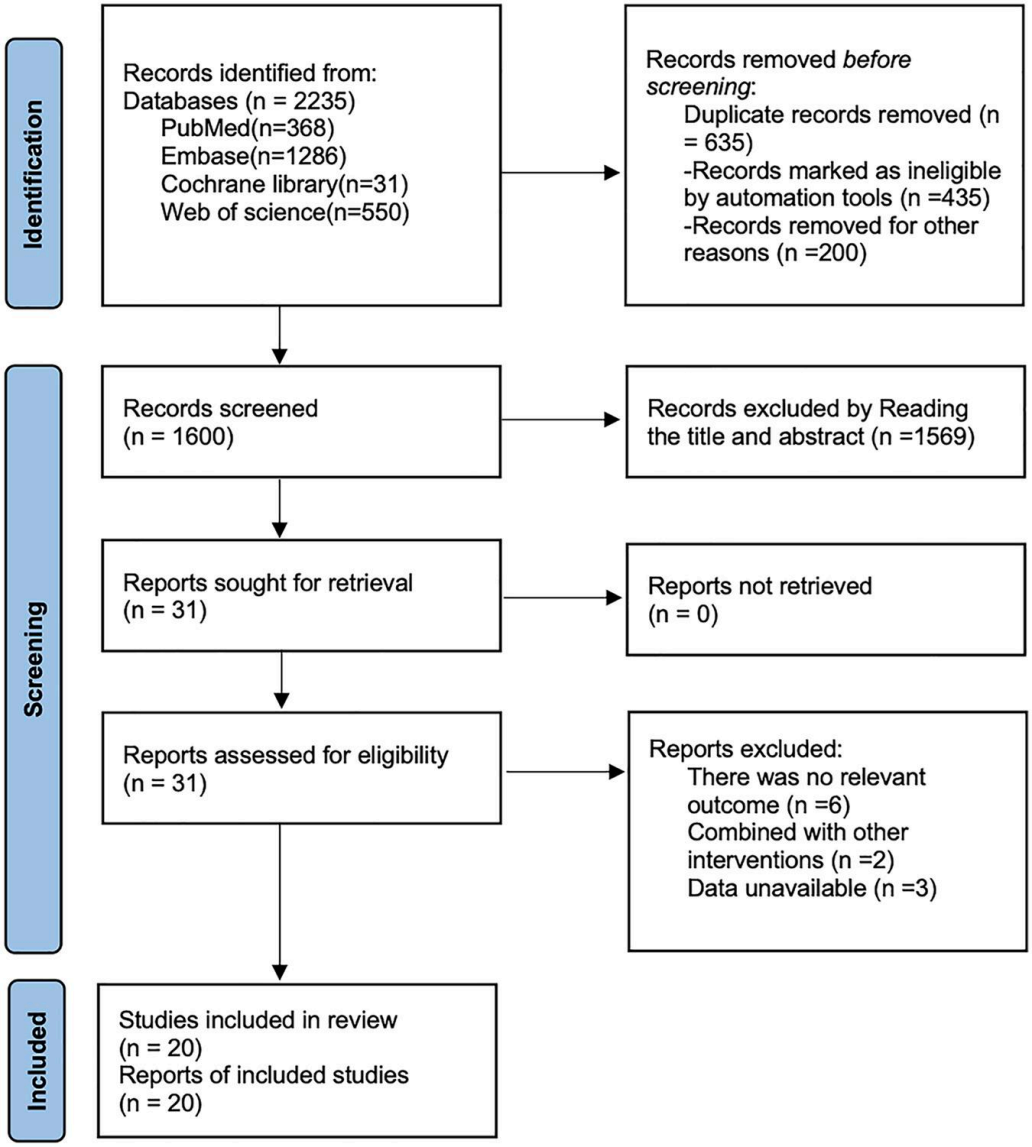
Literature search flow chart.

The initial search yielded 2,235 relevant articles, including 368 from PubMed, 1,286 from Embase, 550 from Web of Science, and 31 from the Cochrane Library. After deduplication using EndNote 21, 635 articles remained. By reviewing titles and abstracts, 1,569 articles were excluded for irrelevance or failure to meet inclusion criteria, leaving 31 articles for full-text screening. Ultimately, 20 studies (17–36) were included. The literature screening process adhered to PRISMA guidelines, as shown in Figure 1.

### Included study characteristics

This study included a total of 20 articles (*N* = 108,343) published between 2000 and 2025. Research origins encompassed Germany, Turkey, Sweden, China, Japan, the United States, the United Kingdom, Denmark, Finland, Switzerland, India, and Poland. Sample sizes ranged from 185 to 2998 cases, with patients' average ages between 43.2 and 55 years. Detailed baseline characteristics are presented in Table 1.

**Table 1:** Basic characteristics of literature.

Study	Year	Country	Study design	Sample size	Gender(M/F)	Mean age	Regression model
Aspinall et al. (17)	2019	UK	Cohort study	10,313	NR	NR	Logistic regression
Aygun et al. (18)	2022	Turkey	Cohort study	875	200/675	49.2	Logistic regression
Bergenfelz et al. (19)	2016	Sweden	Cohort study	5,252	1,050/4,202	49	Logistic regression
Chen et al. (20)	2017	China	Cohort study	3,236	1,044/2,192	45.8	Logistic regression
Dralle et al. (21)	2004	Germany	Cohort study	29,998	6833/22,973	54	Logistic regression
Enomoto et al. (22)	2014	Japan	Cohort study	447	68/375	43.5	Logistic regression
Erbil et al. (23)	2007	Turkey	Case-control	3,250	2,872/378	47	Logistic regression
Godballe et al. (24)	2014	Denmark	Cohort study	6,859	3,419/3,440	52	Logistic regression
Gunn et al. (25)	2020	USA	Cohort study	11,370	2,476/8,894	53	Logistic regression
Han et al. (26)	2024	China	Cohort study	8,340	1,817/6,523	55	Logistic regression
Heikkine et al. (27)	2019	Finland	Cohort study	866	153/713	55	Logistic regression
Joliat et al. (28)	2017	Switzerland	Cohort study	653	200/453	49	Logistic regression
Landerholm et al. (29)	2014	Sweden	Cohort study	1,322	223/1,099	50	Logistic regression
Nayyar et al. (30)	2020	India	Cohort study	228	100/128	55	Logistic regression
Obata et al. (31)	2024	Japan	Cohort study	543	128/415	55	Logistic regression
Staubitz et al. (32)	2020	Germany	Cohort study	4,598	1,056/3,542	54	Logistic regression
Stopenski et al. (33)	2022	USA	Cohort study	11,595	2,522/9,073	52.5	Logistic regression
Tabriz et al. (34)	2024	Germany	Cohort study	1,147	293/854	51	Logistic regression
Thomusch et al. (35)	2000	Germany	Cohort study	7,266	2,266/5,000	51.8	Logistic regression
Wolff et al. (36)	2025	Poland	Cohort study	185	40/145	43.2	Logistic regression

### Quality assessment

This study employed the NOS scoring system. The quality assessment results (Table 2) indicate that 11 articles scored 9 points, 5 studies scored 8 points, and 4 studies scored 7 points. The overall quality of the included studies is classified as high-quality research.

**Table 2:** Nos scores.

Case control									
Study	Is the case definition adequate?	Representativeness of the cases	Determination of control group	Definition of Controls	Comparability of cases and controls based on the design or analysis	Ascertainment of exposure	Same method of ascertainment for cases and controls	Non response	Total scores
Erbil et al. (23)	*	*	*	*	**	*	*	*	9
Cohort study									
Study	Representativeness of the exposed group	Selection of non-exposed groups	Determination of exposure factors	Identification of outcome indicators not yet to be observed at study entry	Comparability of exposed and unexposed groups considered in design and statistical analysis	design and statistical analysis	Adequacy of the study's evaluation of the outcome	Adequacy of follow-up in exposed and unexposed groups	Total scores
Aspinall et al. (17)	*	*	*	*	**	*	*	*	9
Aygun et al. (18)	*	*	*	*	**	*	*	*	9
Bergenfelz et al. (19)	*	*	/	*	**	*	*	*	8
Chen et al. (20)	*	*	/	*	*	*	*	*	7
Dralle et al. (21)	*	*	*	*	**	*	*	*	9
Enomoto et al. (22)	*	*	*	*	**	*	*	*	9
Godballe et al. (24)	*	*	/	*	**	*	*	*	8
Gunn et al. (25)	*	*	/	*	*	*	*	*	7
Han et al. (26)	*	*	*	*	**	*	*	*	9
Heikkine et al. (27)	*	*	*	*	**	*	*	*	9
Joliat et al. (28)	*	*	/	*	**	*	*	*	8
Landerholm et al. (29)	*	*	/	*	*	*	*	*	7
Nayyar et al. (30)	*	*	*	*	**	*	*	*	9
Obata et al. (31)	*	*	*	*	**	*	*	*	9
Staubitz et al. (32)	*	*	*	*	**	*	*	*	9
Stopenski et al. (33)	*	*	/	*	**	*	*	*	8
Tabriz et al. (34)	*	*	/	*	*	*	*	*	7
Thomusch et al. (35)	*	*	*	*	**	*	*	*	9
Wolff et al. (36)	*	*	/	*	**	*	*	*	8

## Meta-analysis results

### Older age

**Figure 2:**
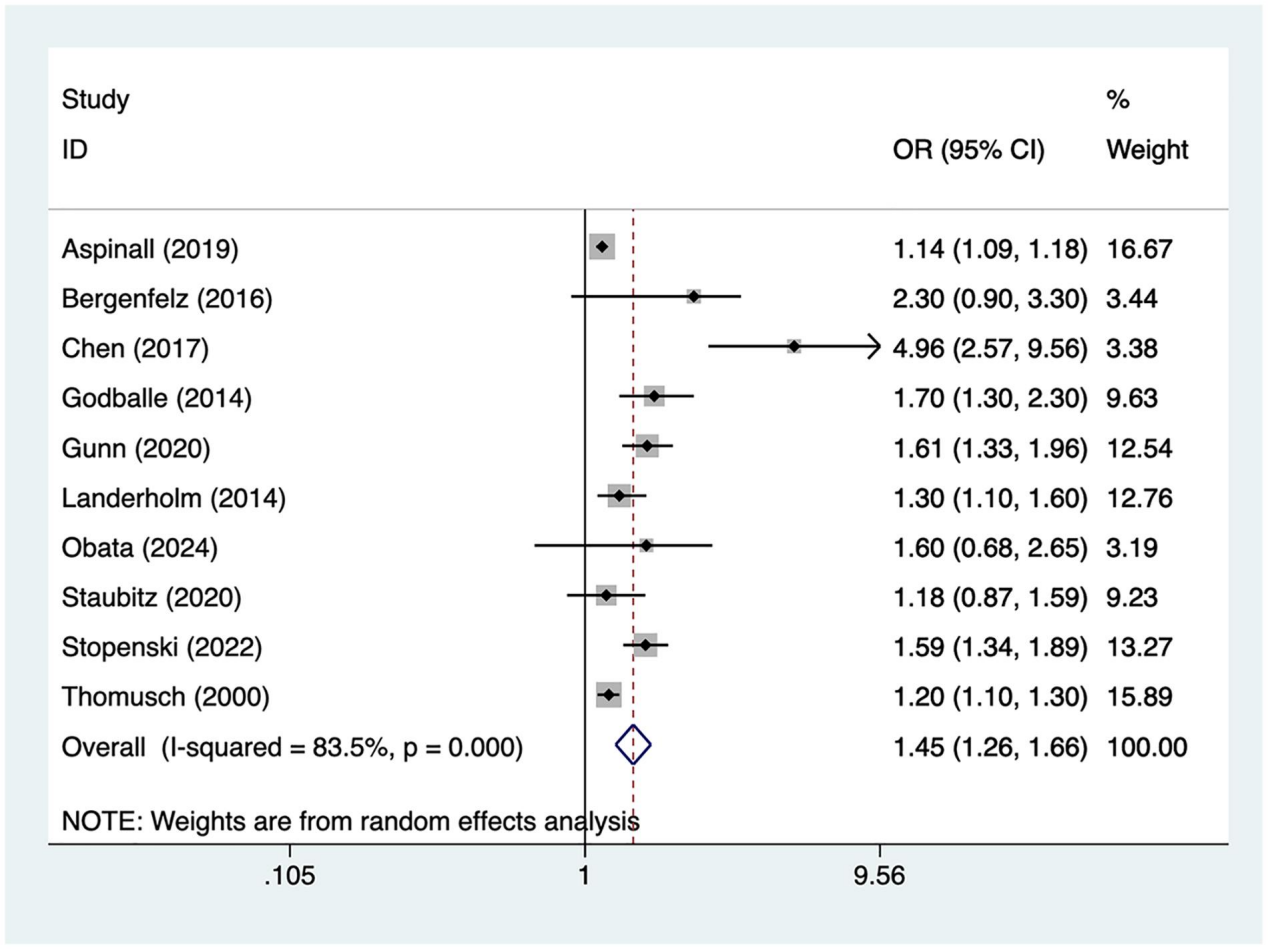
Forest plot of meta-analysis for older age.

10 articles mentioned older age. Heterogeneity testing (*I*² = 83.5%, *P* = 0.001) was conducted using a random-effects model. The results (Figure 2) suggest that older age may be associated with RLNI following thyroid surgery [OR = 1.45, 95% CI (1.26, 1.66)]. Due to substantial heterogeneity, sensitivity analysis was conducted by sequentially excluding studies. Results (Supplementary Figure S1) indicate that this indicator remains robust and is not influenced by any single study.

### Female

**Figure 3:**
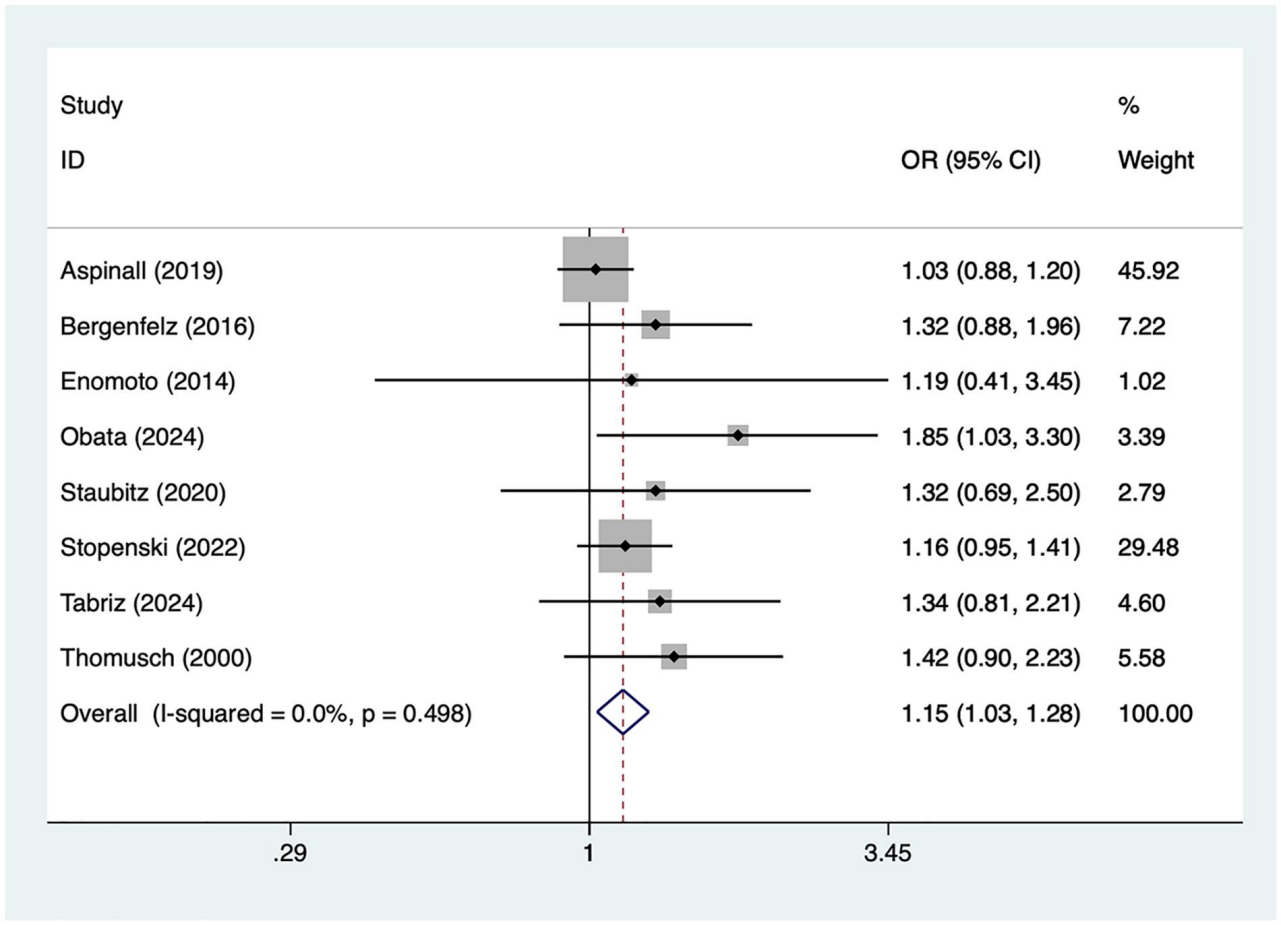
Forest plot of meta-analysis for female.

8 articles mentioned female. Heterogeneity testing (*I*² = 0%, *P* = 0.498) was conducted using a fixed-effects model. The results (Figure 3) suggest that female may be associated with RLNI following thyroid surgery [OR = 1.15, 95% CI (1.03, 1.28)].

### Extended thyroidectomy

**Figure 4:**
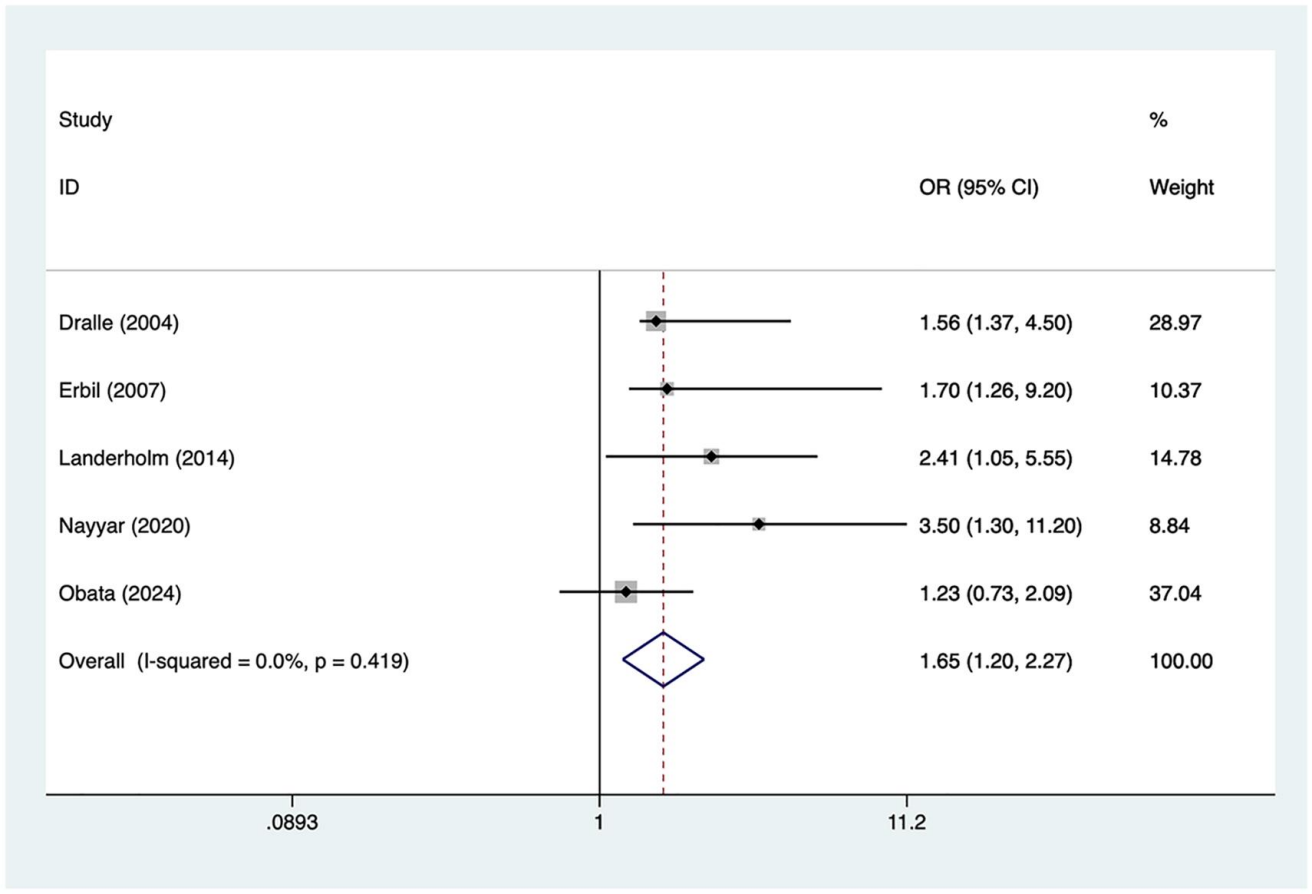
Forest plot of meta-analysis for extended thyroidectomy.

5 articles mentioned extended thyroidectomy. Heterogeneity testing (*I*² = 0%, *P* = 0.419) was conducted using a fixed-effects model. The results (Figure 4) suggest that extended thyroidectomy may be associated with RLNI following thyroid surgery [OR = 1.65, 95% CI (1.20, 2.27)].

### Node dissection

**Figure 5:**
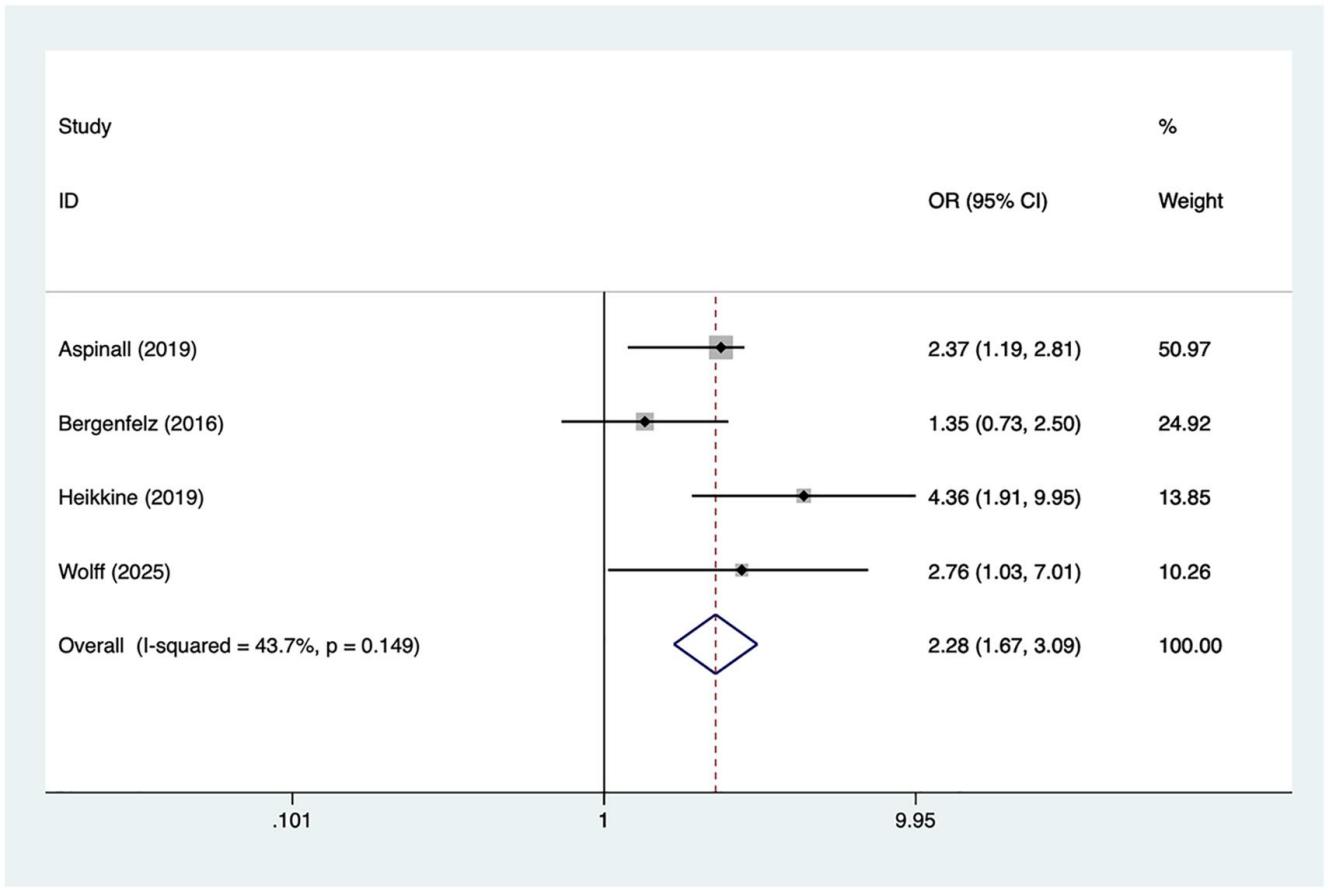
Forest plot of meta-analysis for node dissection.

4 articles mentioned node dissection. Heterogeneity testing (*I*² = 43.7%, *P* = 0.149) was conducted using a fixed-effects model. The results (Figure 5) suggest that node dissection may be associated with RLNI following thyroid surgery [OR = 2.28, 95% CI (1.67, 3.09)].

### Reoperation

**Figure 6:**
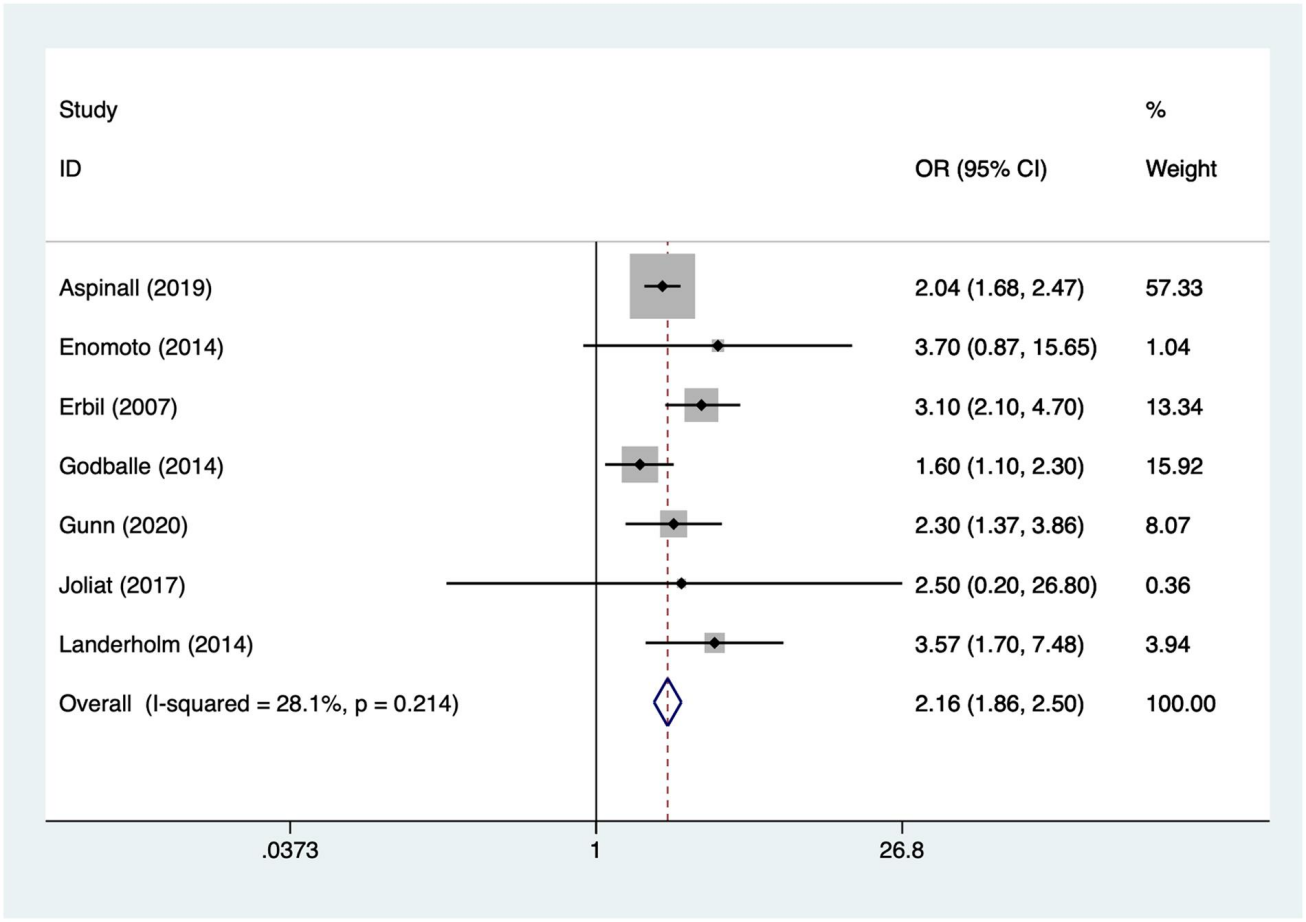
Forest plot of meta-analysis for reoperation.

7 articles mentioned reoperation. Heterogeneity testing (*I*² = 28.1%, *P* = 0.214) was conducted using a fixed-effects model. The results (Figure 6) suggest that reoperation may be associated with RLNI following thyroid surgery [OR = 2.16, 95% CI (1.86, 2.50)].

### Retrosternal goitre

**Figure 7:**
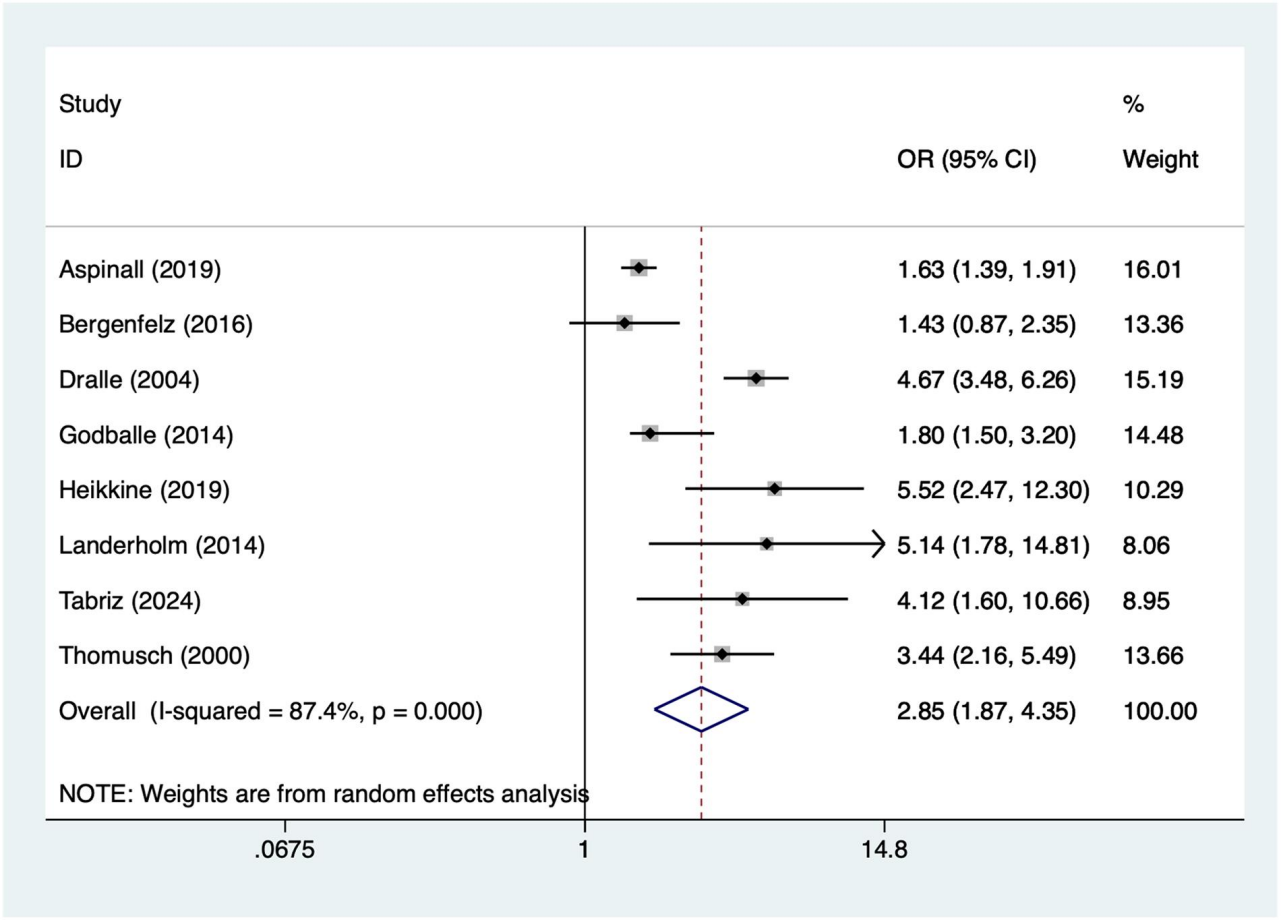
Forest plot of meta-analysis for retrosternal goitre.

10 articles mentioned retrosternal goitre. Heterogeneity testing (*I*² = 87.4%, *P* = 0.001) was conducted using a random-effects model. The results (Figure 7) suggest that retrosternal goitre may be associated with RLNI following thyroid surgery [OR = 2.85, 95% CI (1.87, 4.35)]. Due to substantial heterogeneity, sensitivity analysis was conducted by sequentially excluding studies. Results (Supplementary Figure S2) indicate that this indicator remains robust and is not influenced by any single study.

### Lack of neuromonitoring

**Figure 8:**
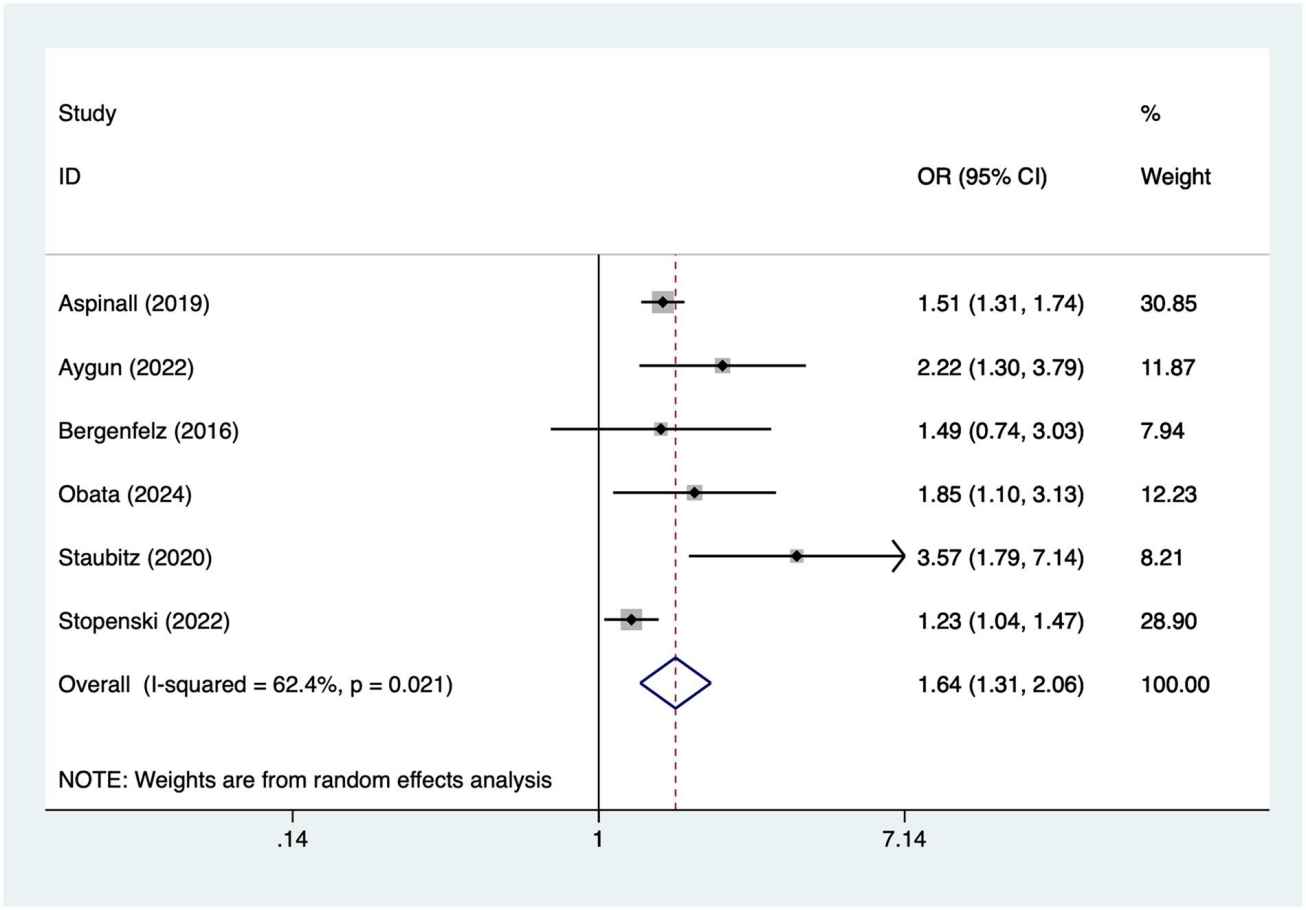
Forest plot of meta-analysis for lack of neuromonitoring.

6 articles mentioned lack of neuromonitoring. Heterogeneity testing (*I*² = 62.4%, *P* = 0.021) was conducted using a random-effects model. The results (Figure 8) suggest that lack of neuromonitoring may be associated with RLNI following thyroid surgery [OR = 1.64, 95% CI (1.31, 2.06)]. Due to substantial heterogeneity, sensitivity analysis was conducted by sequentially excluding studies. Results (Supplementary Figure S3) indicate that this indicator remains robust and is not influenced by any single study.

### Publication bias

This study employed funnel plots and Egger's test to detect publication bias. Results (Supplementary Figures S4–S10) suggest a higher likelihood of publication bias for older age (*P* = 0.002) and female gender (*P* = 0.023). Conversely, publication bias was less likely to exist for extended thyroidectomy (*P* = 0.231), node dissection (*P* = 0.189), reoperation (*P* = 0.298), retrosternal goiter (*P* = 0.170), and lack of neuromonitoring (*P* = 0.154). Perform trim-and-fill adjustment for indicators exhibiting publication bias. Results (Supplementary Figures S11–S12) indicate that despite the presence of publication bias, the findings of this study remain robust.

## Discussion

This study comprehensively explored and quantitatively analyzed the primary risk factors for RLNI following thyroid surgery. Our findings indicate that multiple factors are significantly associated with the occurrence of RLNI, including age, gender, surgical type, recurrent goiter, and the absence of intraoperative nerve monitoring.

In this study, older age was identified as an independent risk factor for RLNI. This finding indicates that the risk of RLNI increases significantly with advancing age. Older patients typically experience physiological decline, including reduced muscle elasticity, stiffened vascular walls, and diminished tissue immune responses (37). These changes may impair soft tissue recovery during surgery, thereby increasing the risk of nerve injury.

Additionally, the anatomical structure of the thyroid gland often changes in elderly patients, particularly exhibiting increased volume, harder texture, and even calcification, which complicates surgical manipulation (38). Among thyroid cancer patients, the elderly group faces an especially heightened risk of nerve injury, likely due to the cancer's inherent invasiveness and extensive surrounding tissue adhesions. Postoperative recovery is slower in elderly patients, and the likelihood of nerve recovery after injury is lower, potentially increasing the persistence and severity of RLNI (39). Therefore, for elderly patients, especially those requiring complex procedures, consideration should be given to employing intraoperative nerve monitoring techniques and utilizing minimally invasive surgical approaches to reduce the incidence of nerve injury.

Our research indicates that women face a higher risk of RLNI following thyroid surgery compared to men. This gender difference may stem from female thyroid anatomy and hormonal levels. Studies show that women's thyroids are generally larger and softer than men's, potentially leading to greater deformation during surgery or more frequent contact with the recurrent laryngeal nerve (40). Additionally, female thyroids exhibit richer vascularity, increasing susceptibility to hematoma or edema formation, which further elevates nerve injury risk. Hormonal levels, particularly estrogen, may also influence nerve repair capacity to some extent (41). Some studies suggest estrogen offers protective effects for nerves; however, during surgical trauma, this protective action may paradoxically hinder adaptive nerve recovery. This could be one reason for poorer postoperative nerve injury recovery in female patients.

Extensive thyroid resection is a significant risk factor for RLNI. This surgical approach typically requires a larger resection margin and greater exposure of tissues and structures during the procedure, increasing the risk of recurrent laryngeal nerve injury. In extended surgeries, the anatomical structures surrounding the thyroid gland are more complex, making adjacent nerves and vessels susceptible to traction, compression, or thermal injury (10). Furthermore, extended thyroidectomy is associated with longer operative times and higher intraoperative blood flow, which also heightens the risk of nerve injury (42). Particularly in thyroid cancer cases, tumors may have invaded or formed tight adhesions with the recurrent laryngeal nerve and its blood supply, making nerve injury more likely during resection. Consequently, intraoperative nerve monitoring is critical for such complex procedures, enabling real-time assessment of nerve function to prevent unnecessary damage.

Our findings indicate that lymph node dissection is a factor closely associated with the occurrence of RLNI. Lymph node dissection procedures typically involve larger resection areas and more complex anatomical structures, increasing exposure to and manipulation difficulty of the recurrent laryngeal nerve (43). Lymph node dissection, particularly when performed in the central region, often requires careful identification and avoidance of critical neural structures. Another potential mechanism for lymph node dissection-related RLNI may be related to the duration of intraoperative manipulation. Prolonged surgical time and sustained anatomical dissection may lead to hypoxia or insufficient blood supply to neural tissue, thereby increasing the risk of nerve injury (44). For patients undergoing lymph node dissection, the use of intraoperative nerve monitoring technology allows real-time assessment of recurrent laryngeal nerve function, helping to avoid unnecessary injury.

The risk of RLNI is markedly elevated in patients undergoing reoperation. These patients often present with existing postoperative scar tissue and adhesions, making nerves more vulnerable to injury. In such cases, altered anatomical landmarks and residual surgical traces complicate nerve exposure (45). Particularly for patients with prior thyroidectomy, re-excision may necessitate complex surgical maneuvers, potentially leading to unavoidable nerve traction or transection. Furthermore, recurrent disease may exacerbate the risk of nerve injury, with malignant tumors posing a particularly high risk (46). For such patients, detailed preoperative assessment is essential to thoroughly understand the anatomical structures and extent of the lesion within the surgical field. Minimally invasive techniques should be prioritized whenever feasible to minimize the probability of nerve injury.

Retrosternal goiter is considered a significant risk factor for RLNI. Due to its deep location and proximity to the recurrent laryngeal nerve and its blood supply, resection of retrosternal goiter necessitates a larger surgical incision and more complex manipulation, increasing the risk of both direct and indirect injury to the recurrent laryngeal nerve (47). Furthermore, retrosternal goiters often present with prolonged compressive symptoms, potentially leading to diminished nerve function (48). This renders the nerves more vulnerable and susceptible to injury during surgery.

The absence of intraoperative nerve monitoring has been established as an independent risk factor for RLNI. Intraoperative nerve monitoring technology enables real-time assessment of recurrent laryngeal nerve function, allowing early detection of nerve injury signs and guiding surgeons to avoid unnecessary damage (49). Without nerve monitoring, surgeons often rely solely on surgical experience and anatomical knowledge, which may lead to missed or misdiagnosed nerve injuries, particularly in complex procedures or cases with significant anatomical variations. Therefore, promoting the use of intraoperative nerve monitoring technology is crucial for reducing the incidence of RLNI.

## Strengths and limitations

The primary strength of this study lies in its application of systematic review and meta-analysis methodologies, integrating data from multiple high-quality studies to achieve robust statistical power and a high level of evidence. By synthesizing findings across numerous investigations, this research accurately identifies key risk factors for recurrent laryngeal nerve injury following thyroid surgery and quantifies their effect sizes. Furthermore, stringent inclusion criteria were employed to ensure the quality and consistency of all included studies. Sensitivity analyses and subgroup analyses further validated the robustness of the findings, mitigating the potential influence of individual studies on overall results. This enables us to draw more reliable and broadly applicable conclusions, providing valuable evidence-based support for risk assessment and management in clinical practice.

Although this study offers a systematic analysis of risk factors for recurrent laryngeal nerve injury following thyroid surgery, several limitations exist. First, the majority of included studies were retrospective, potentially introducing selection bias and incomplete data, which limits the generalizability of conclusions. Second, although sensitivity analyses addressed sources of heterogeneity, differences in surgical techniques, patient characteristics, and diagnostic criteria for nerve injury across studies may still affect the consistency and reliability of results. Finally, this study did not include all potential risk factors, such as surgeon experience and specific patient pathological conditions, which may also influence nerve injury occurrence. Therefore, future research should further explore and validate these factors to enhance the accuracy of risk assessment.

## Clinical significance

This study identified multiple risk factors associated with RLNI following thyroid surgery, including older age, female gender, extended thyroidectomy, and lymph node dissection. These findings assist clinicians in conducting preoperative risk assessments and implementing personalized surgical strategies. Particularly for high-risk patients, such as the elderly or those requiring complex procedures, the use of intraoperative nerve monitoring technology can effectively reduce the incidence of RLNI, thereby enhancing surgical safety and improving the quality of postoperative recovery. Therefore, understanding these risk factors and appropriately utilizing nerve monitoring are crucial for improving the prognosis of thyroid surgery.

## Conclusion

This study indicates that older age, female gender, extended thyroidectomy, lymph node dissection, reoperation, retrosternal goiter, and absence of nerve monitoring mya be independent risk factors for recurrent laryngeal nerve injury following thyroid surgery. Clinicians should assess these risk factors based on individual patient circumstances and implement nerve-sparing techniques during surgery whenever feasible. Particularly in complex procedures, the use of intraoperative nerve monitoring may significantly reduce the incidence of recurrent laryngeal nerve injury.

## Data Availability

The original contributions presented in the study are included in the article/Supplementary Material, further inquiries can be directed to the corresponding author.
