# Supplementary material for: Risk factors for recurrent laryngeal nerve injury following thyroid surgery: a systematic review and meta-analysis
Source: Front Surg. 2026 Jan 7;12:1731701. doi: 10.3389/fsurg.2025.1731701 (PMC12819817; doi:10.3389/fsurg.2025.1731701)
Supplement: Supplementary file 1 [file Datasheet1.docx]

Table S1 Search strategy

((("Recurrent Laryngeal Nerve Injuries"[Mesh]) OR ((((((((((((((((((((((((((((((((((((((((Recurrent Laryngeal Nerve Injuries[Title/Abstract]) OR (Recurrent Laryngeal Nerve Injury[Title/Abstract])) OR (Recurrent Laryngeal Nerve Trauma[Title/Abstract])) OR (Recurrent Laryngeal Neuropathy, Traumatic[Title/Abstract])) OR (Recurrent Laryngeal Nerve Contusion[Title/Abstract])) OR (Recurrent Laryngeal Nerve Transection[Title/Abstract])) OR (Vocal Cord Paralysis[Title/Abstract])) OR (Paralyses, Vocal Cord[Title/Abstract])) OR (Paralysis, Vocal Cord[Title/Abstract])) OR (Vocal Cord Paralyses[Title/Abstract])) OR (Total Vocal Cord Paralysis[Title/Abstract])) OR (Vocal Cord Palsy[Title/Abstract])) OR (Palsies, Vocal Cord[Title/Abstract])) OR (Palsy, Vocal Cord[Title/Abstract])) OR (Vocal Cord Palsies[Title/Abstract])) OR (Vocal Fold Palsy[Title/Abstract])) OR (Palsies, Vocal Fold[Title/Abstract])) OR (Palsy, Vocal Fold[Title/Abstract])) OR (Vocal Fold Palsies[Title/Abstract])) OR (Laryngeal Paralysis[Title/Abstract])) OR (Laryngeal Paralyses[Title/Abstract])) OR (Paralyses, Laryngeal[Title/Abstract])) OR (Paralysis, Laryngeal[Title/Abstract])) OR (Laryngeal Nerve Palsy, Recurrent[Title/Abstract])) OR (Recurrent Laryngeal Nerve Palsy[Title/Abstract])) OR (Acquired Vocal Cord Palsy[Title/Abstract])) OR (Bilateral Vocal Cord Paresis[Title/Abstract])) OR (Paralysis, Unilateral, Vocal Cord[Title/Abstract])) OR (Paralysis, Vocal Cord, Unilateral[Title/Abstract])) OR (Unilateral Paralysis, Vocal Cord[Title/Abstract])) OR (Unilateral Vocal Cord Paralysis[Title/Abstract])) OR (Vocal Cord Paralysis, Unilateral[Title/Abstract])) OR (Partial Paralysis (Paresis) Vocal Cords[Title/Abstract])) OR (Vocal Cord Paresis[Title/Abstract])) OR (Pareses, Vocal Cord[Title/Abstract])) OR (Paresis, Vocal Cord[Title/Abstract])) OR (Vocal Cord Pareses[Title/Abstract])) OR (Unilateral Vocal Cord Paresis[Title/Abstract])) OR (Congenital Vocal Cord Palsy[Title/Abstract])) OR (Vocal Cord Palsy, Congenital[Title/Abstract]))) AND ((thyroid surgery[MeSH Terms]) OR (((thyroid surgery[Title/Abstract]) OR (thyroid[Title/Abstract])) OR (Thyroidectomy[Title/Abstract])))) AND (("Risk Factors"[Mesh]) OR (((((((((((((((((((Risk Factors[Title/Abstract]) OR (Factor, Risk[Title/Abstract])) OR (Risk Factor[Title/Abstract])) OR (Population at Risk[Title/Abstract])) OR (Populations at Risk[Title/Abstract])) OR (Risk Scores[Title/Abstract])) OR (Risk Score[Title/Abstract])) OR (Score, Risk[Title/Abstract])) OR (Risk Factor Scores[Title/Abstract])) OR (Risk Factor Score[Title/Abstract])) OR (Score, Risk Factor[Title/Abstract])) OR (Health Correlates[Title/Abstract])) OR (Correlates, Health[Title/Abstract])) OR (Social Risk Factors[Title/Abstract])) OR (Factor, Social Risk[Title/Abstract])) OR (Factors, Social Risk[Title/Abstract])) OR (Risk Factor, Social[Title/Abstract])) OR (Risk Factors, Social[Title/Abstract])) OR (Social Risk Factor[Title/Abstract])))


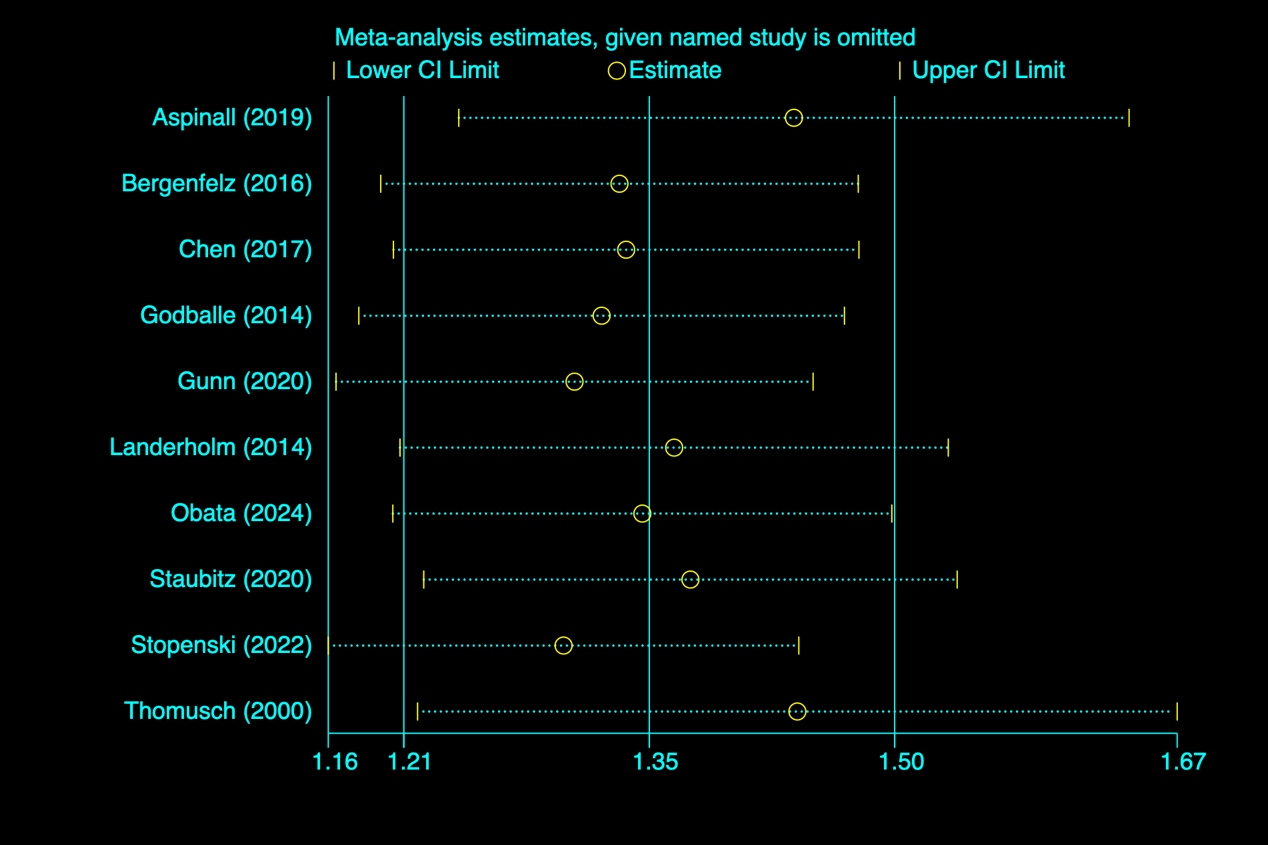


Figure S1 Sensitivity analysis of the older age


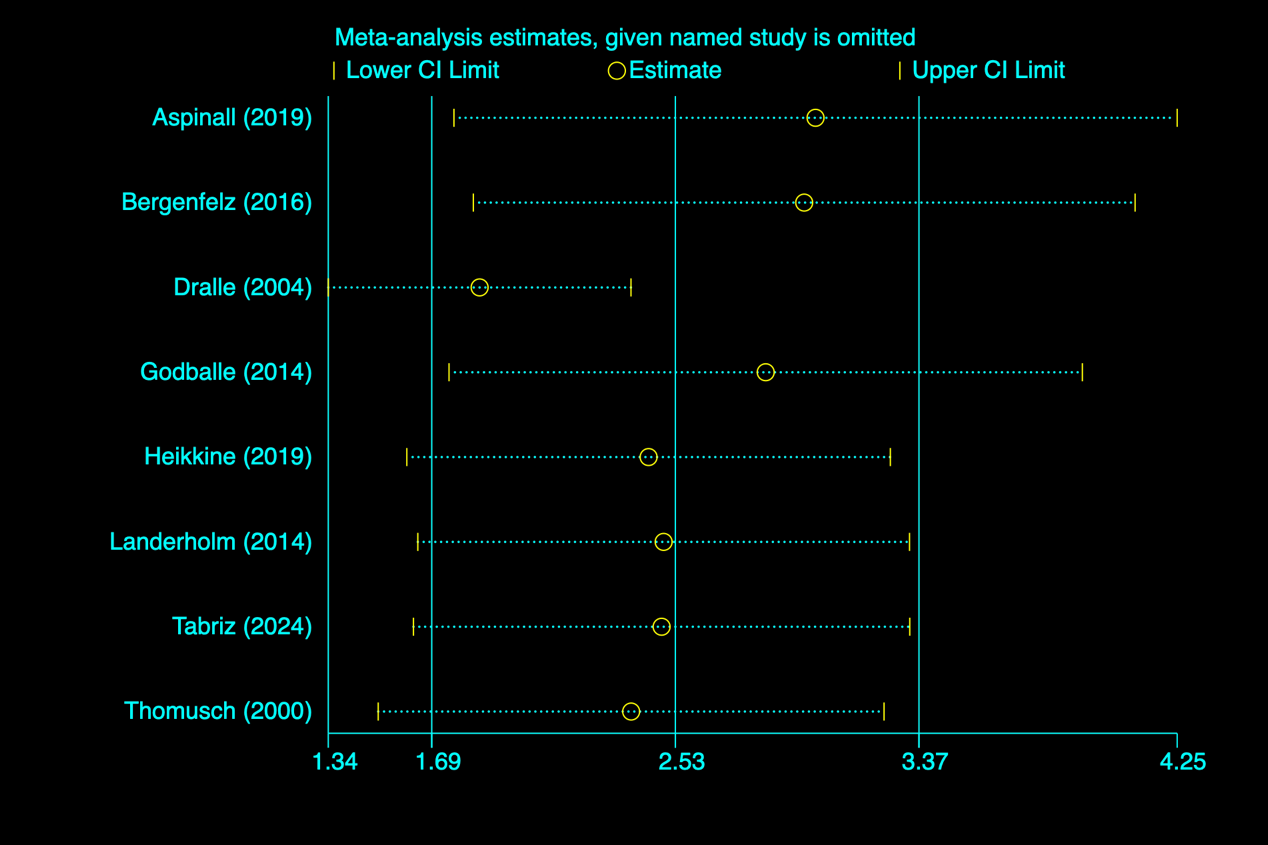


Figure S2 Sensitivity analysis of the retrosternal goitre


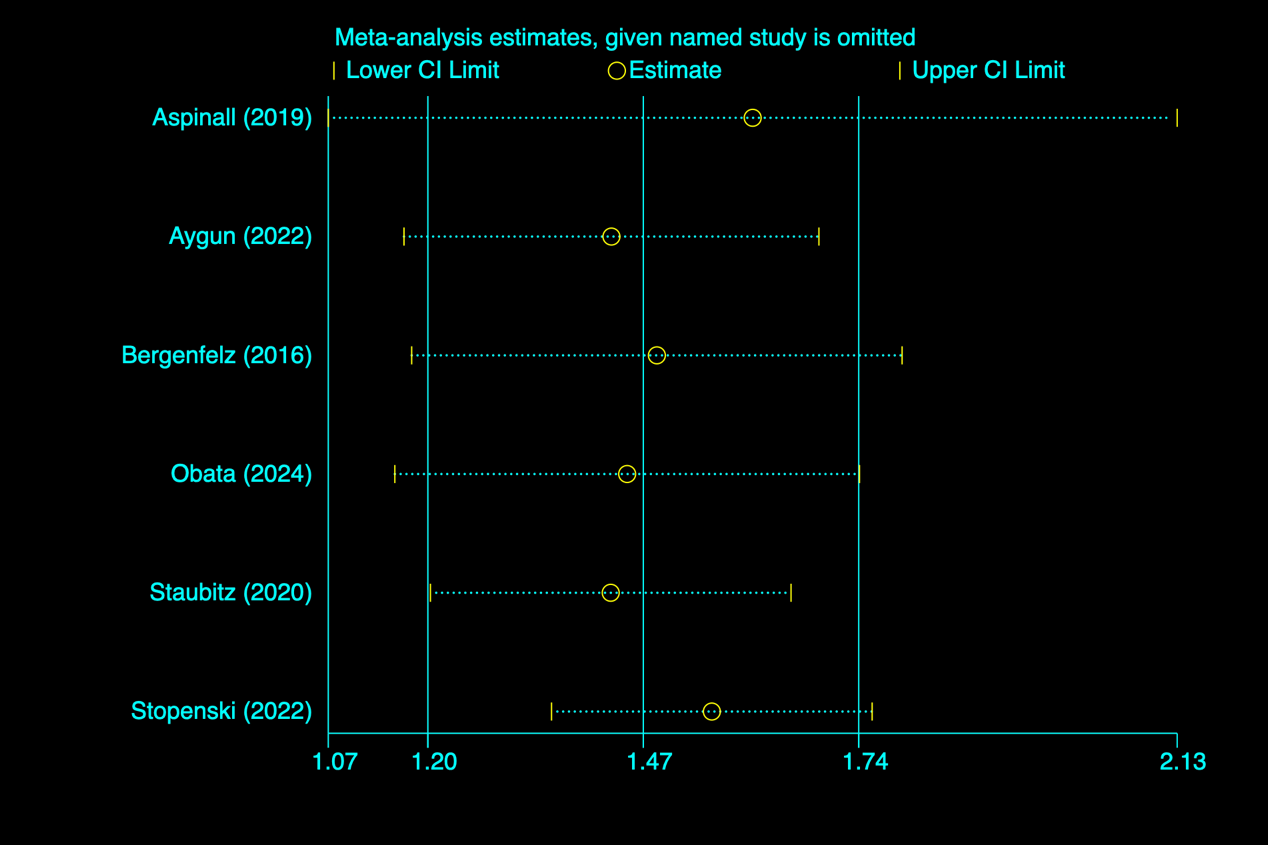


Figure S3 Sensitivity analysis of the lack of neuromonitoring


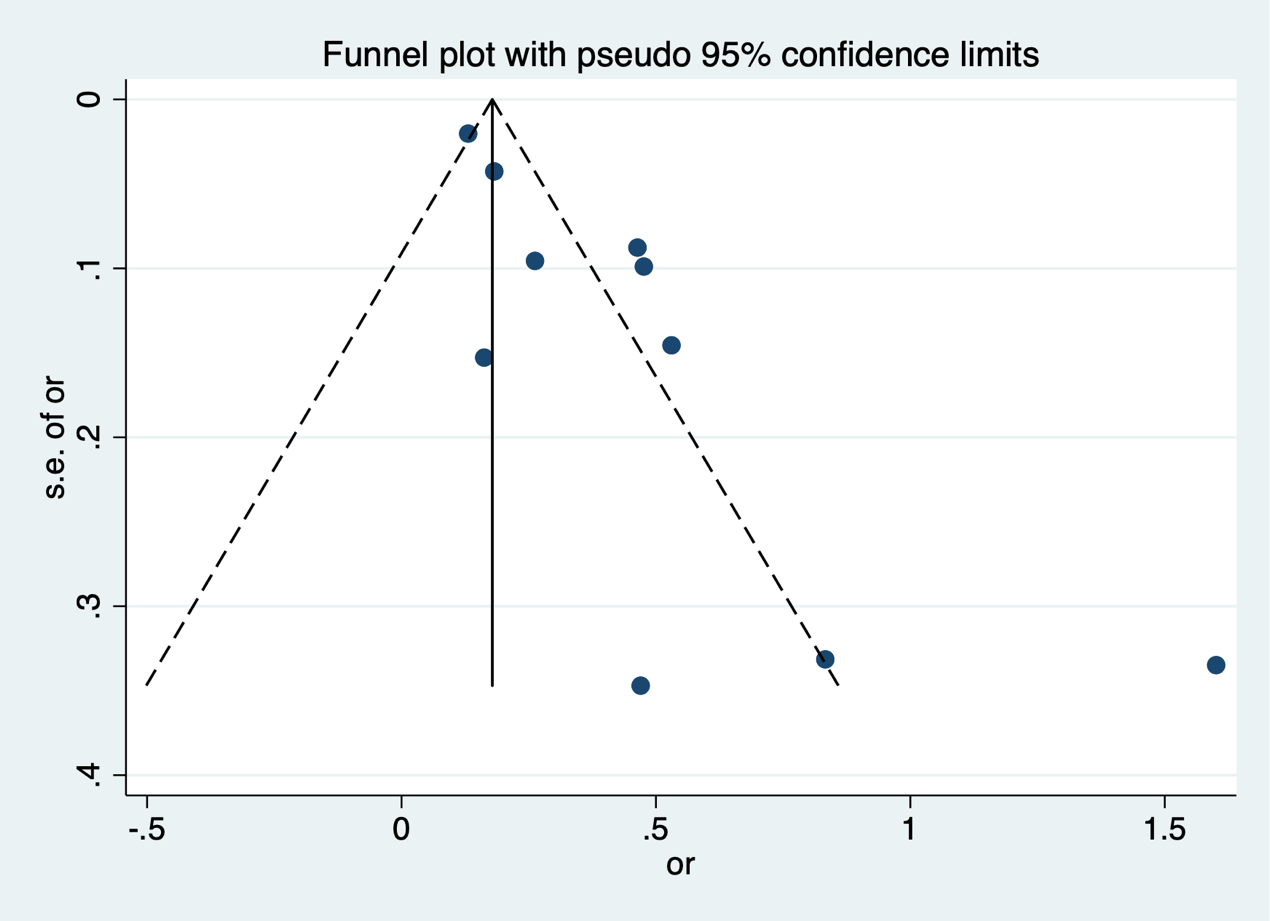


Figure S4 Funnel plot for meta-analysis of older age


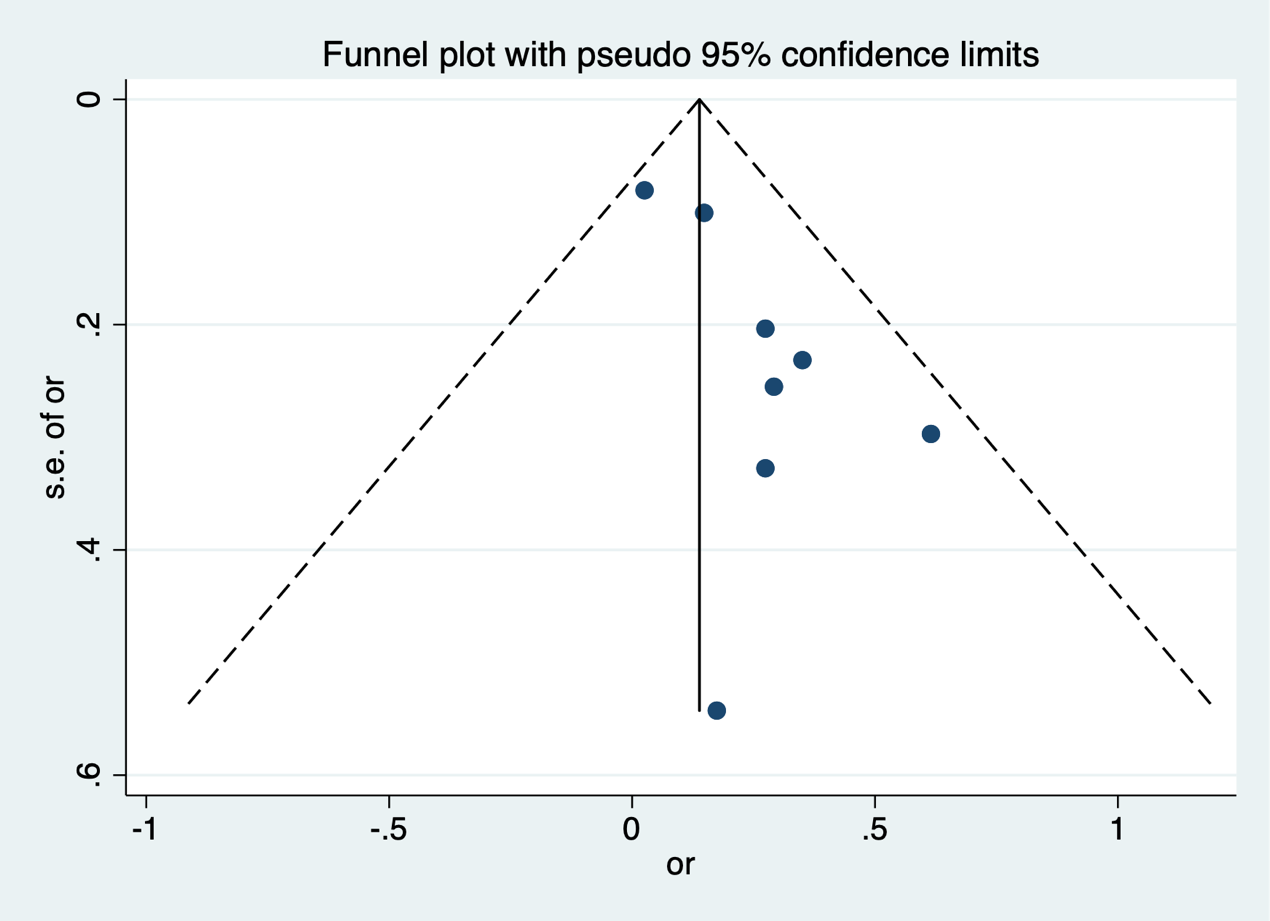


Figure S5 Funnel plot for meta-analysis of female


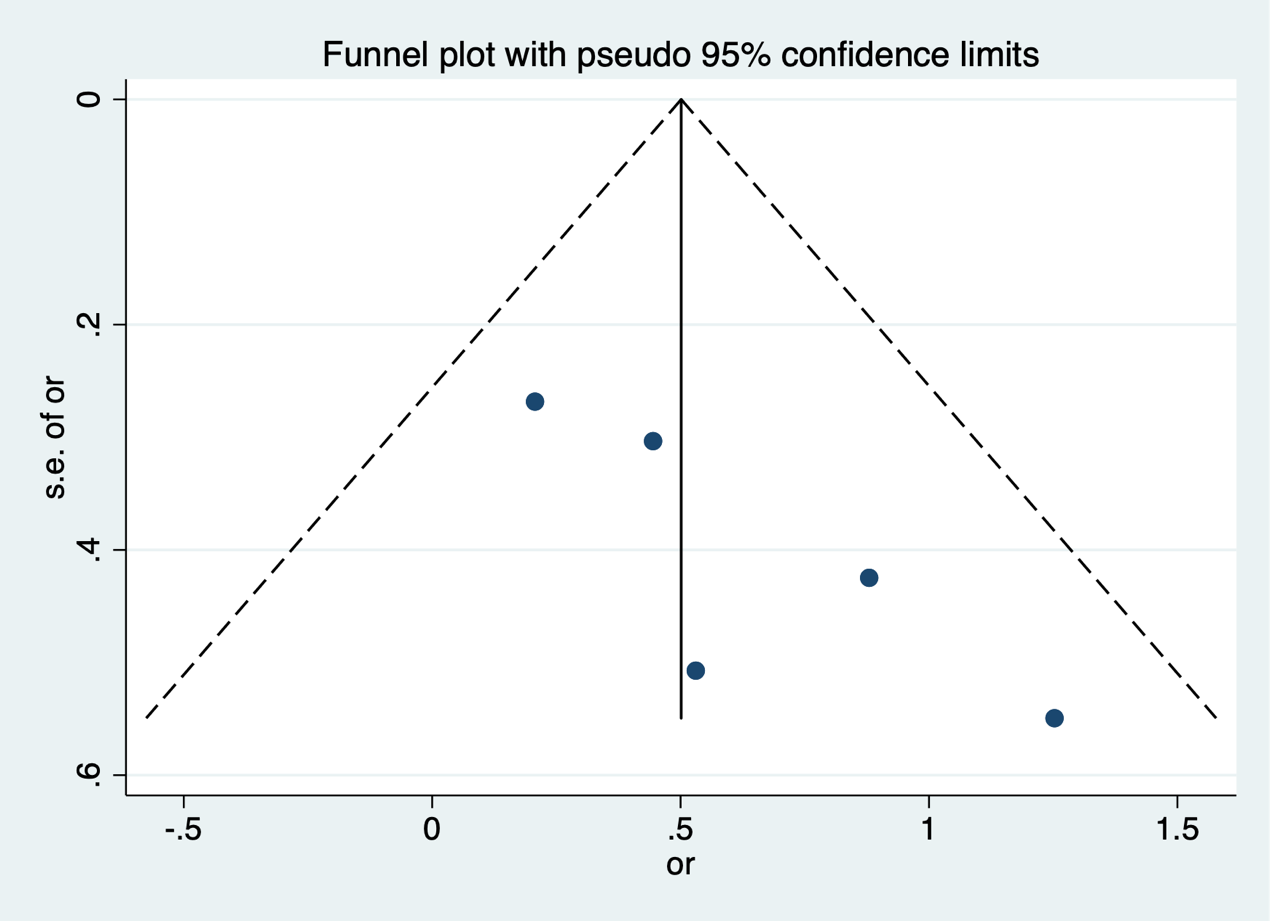


Figure S6 Funnel plot for meta-analysis of extended thyroidectomy


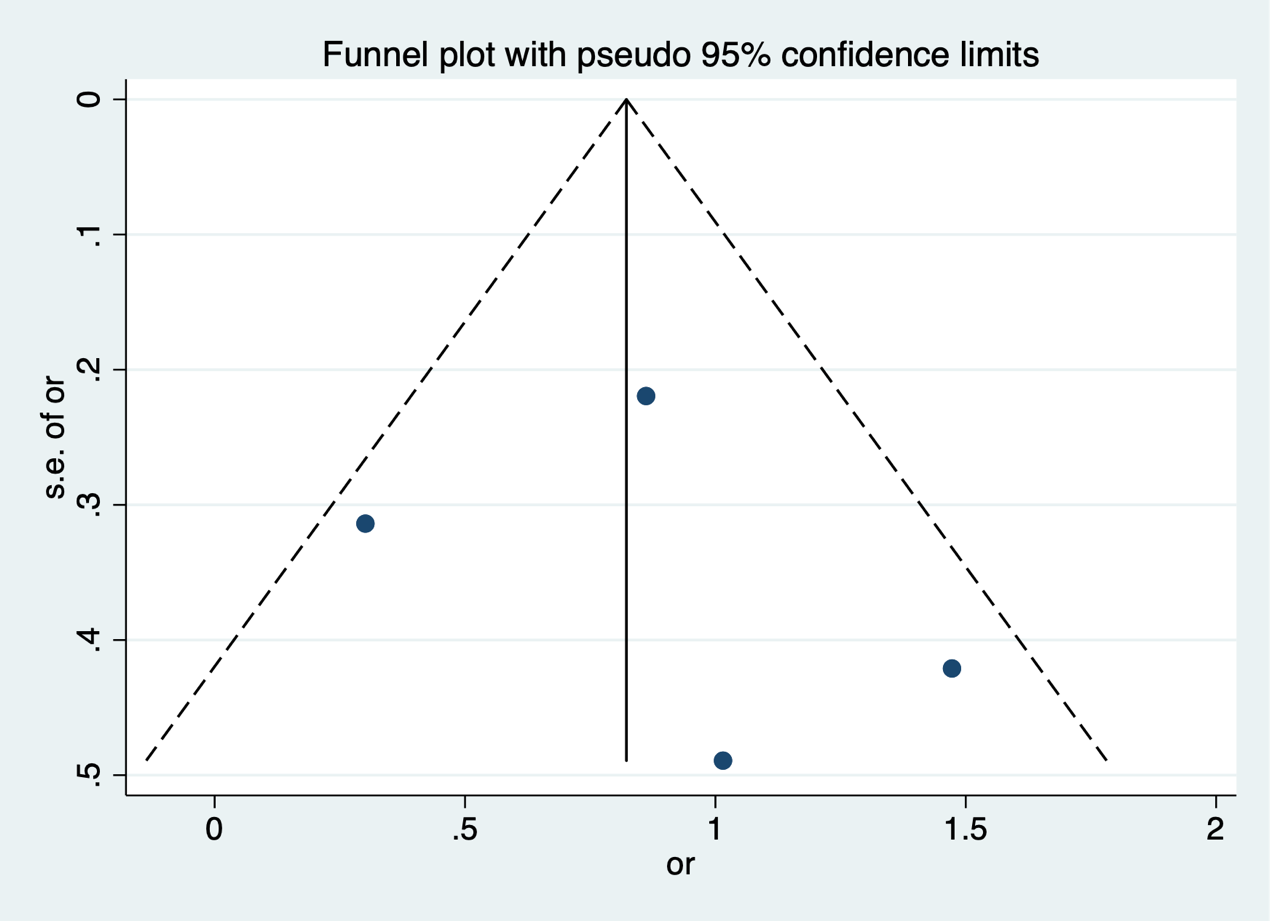


Figure S7 Funnel plot for meta-analysis of node dissection


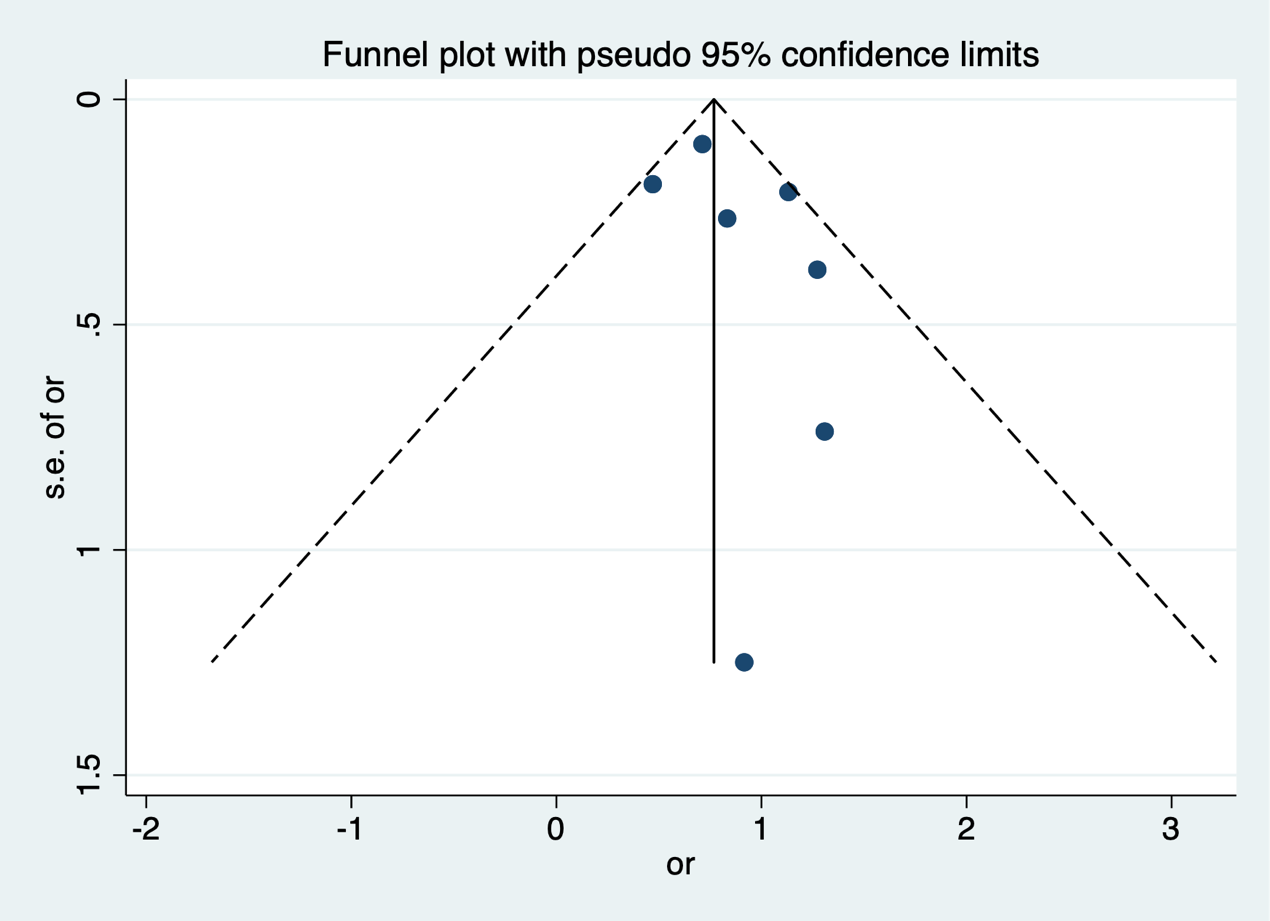


Figure S8 Funnel plot for meta-analysis of reoperation


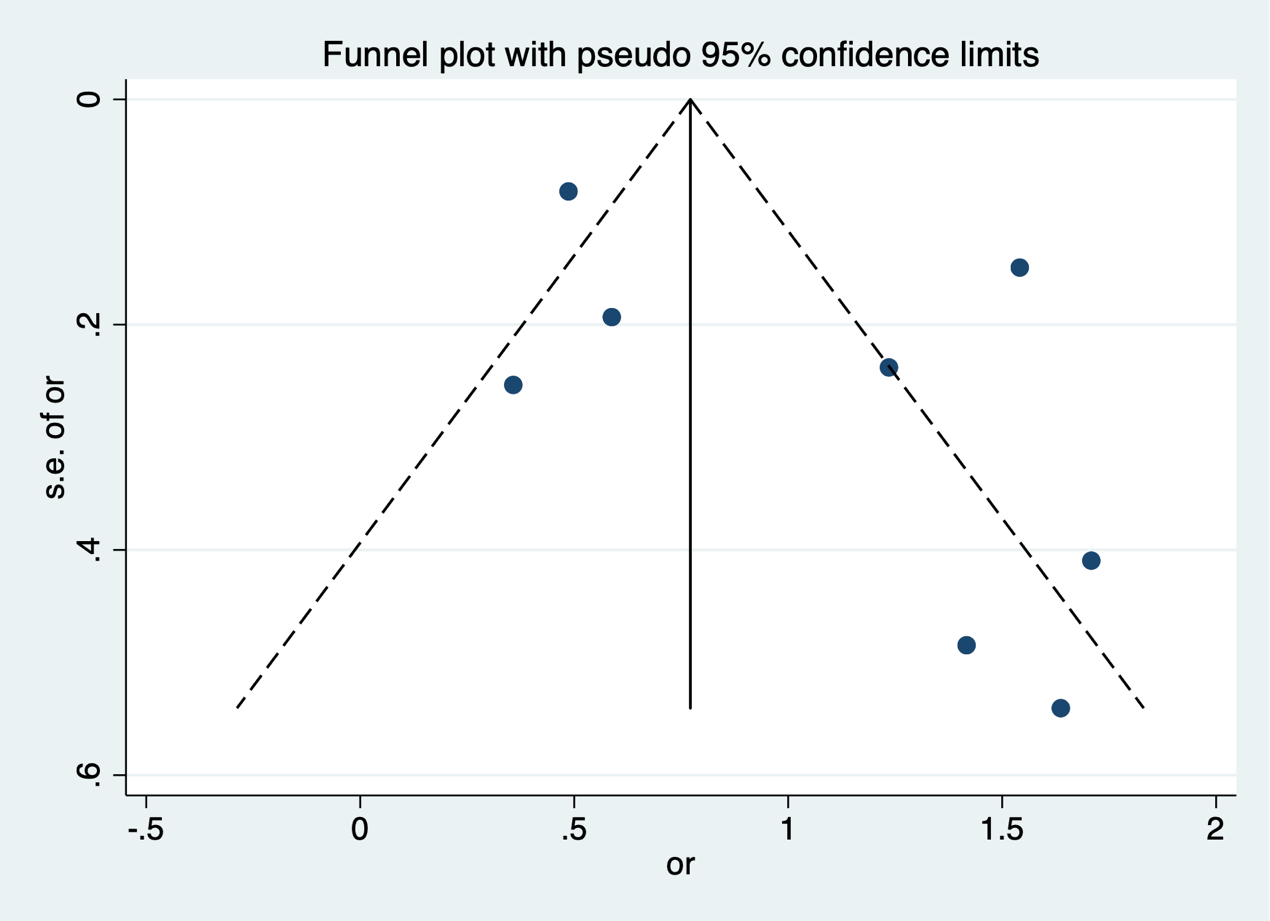


Figure S9 Funnel plot for meta-analysis of retrosternal goitre


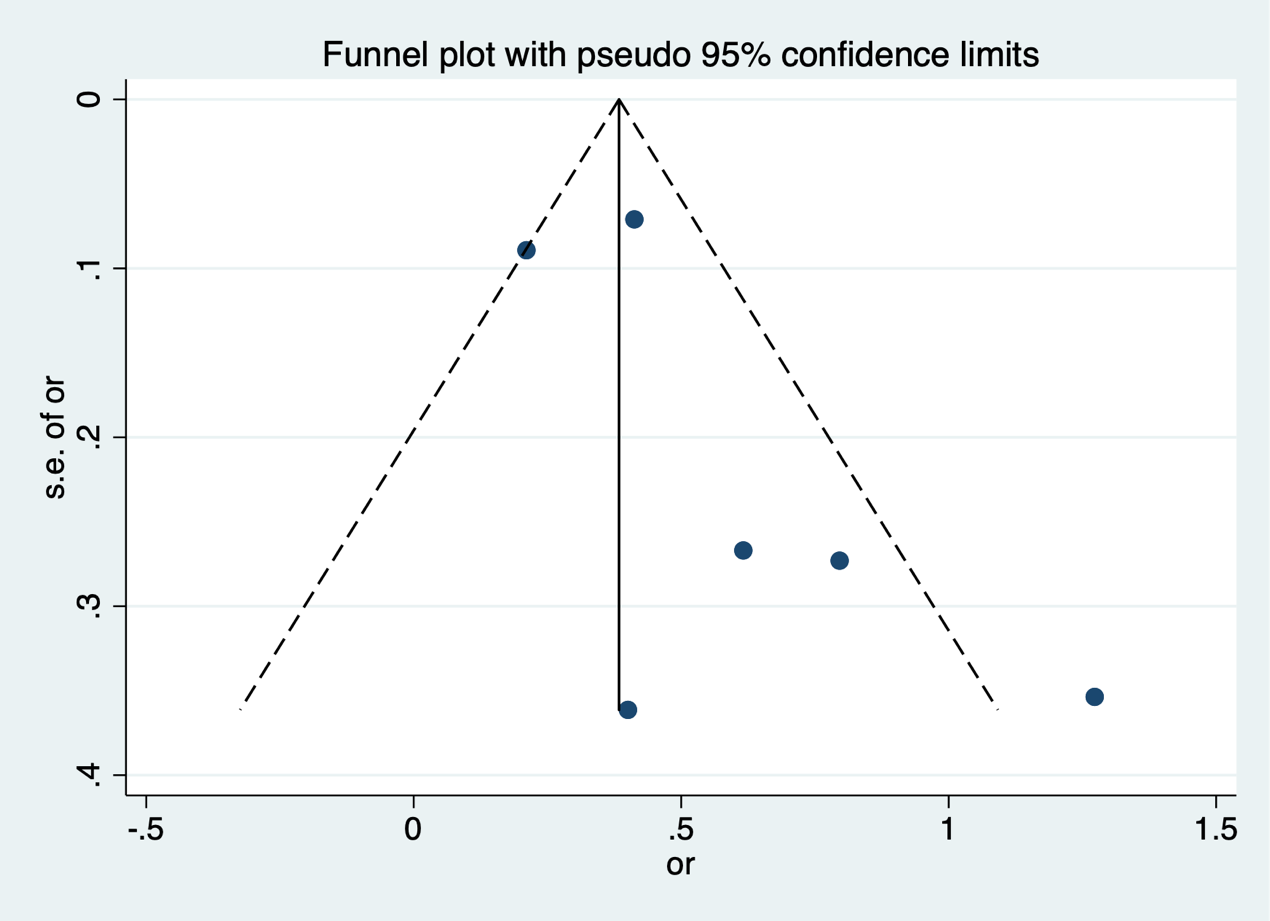
 Figure S10 Funnel plot for meta-analysis of lack of neuromonitoring


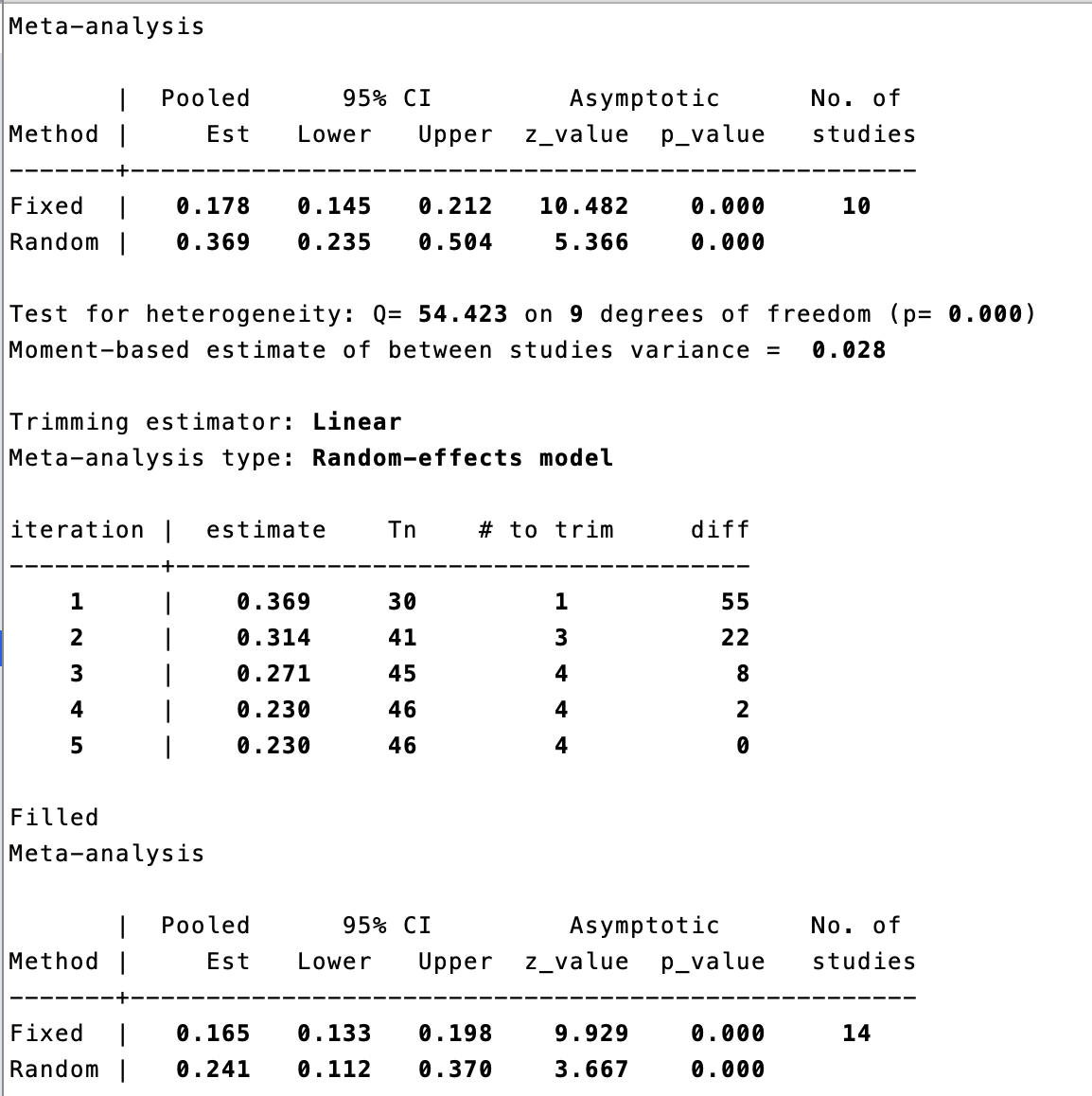


Figure S11 trim-and-fill results for older age


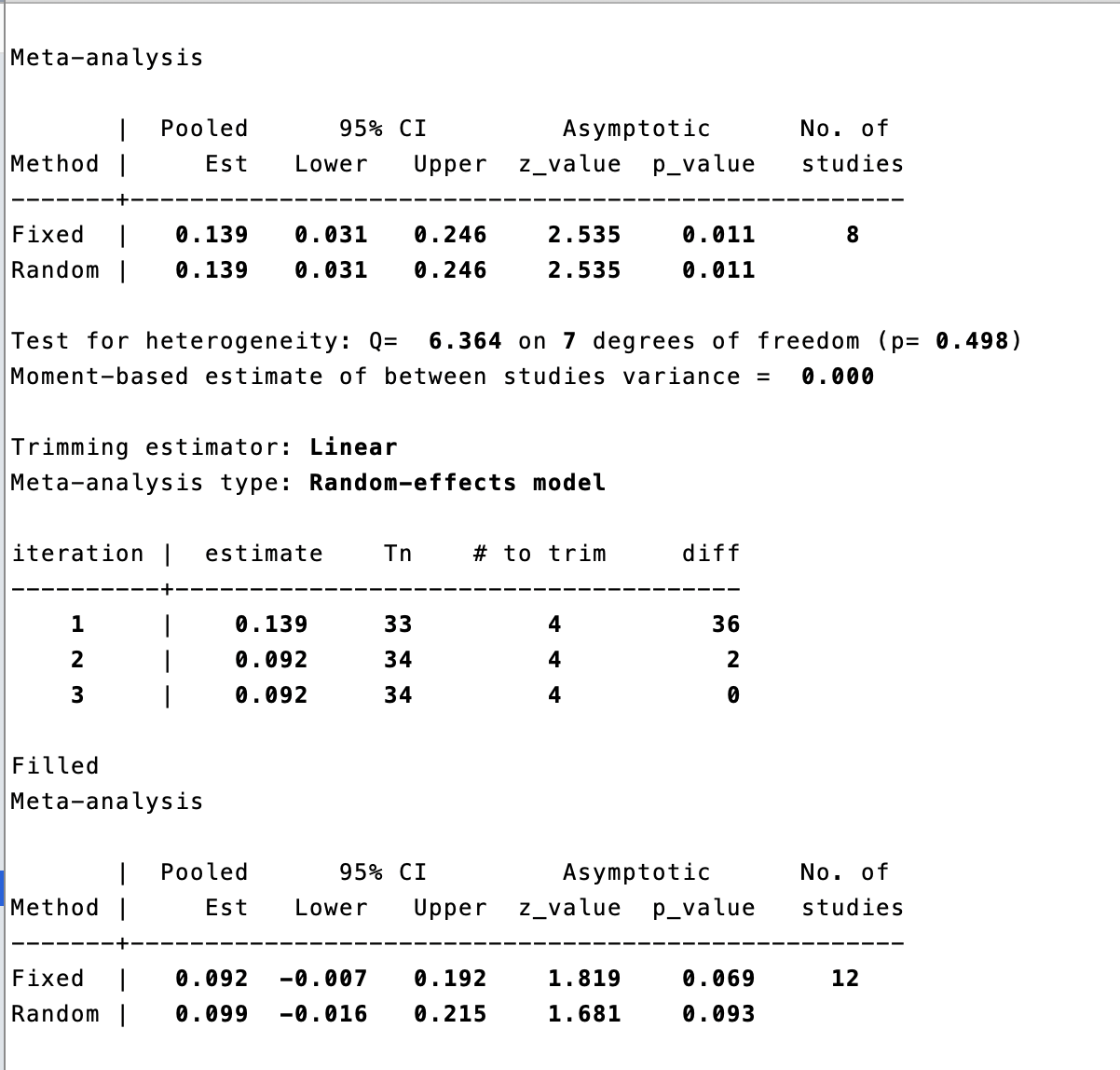


Figure S11 trim-and-fill results for female
