# Supplementary material for: Risk factors for recurrent laryngeal nerve injury following thyroid surgery: a systematic review and meta-analysis
Source: Front Surg. 2026 Jan 7;12:1731701. doi: 10.3389/fsurg.2025.1731701 (PMC12819817; doi:10.3389/fsurg.2025.1731701)
Supplement: Supplementary file 2 [file Table1.docx]

| study | year | country | study design | sample size | gender(M/F) | mean age | Regression model |
| --- | --- | --- | --- | --- | --- | --- | --- |
| Aspinall | 2019 | UK | cohort study | 10313 | NR | NR | logistic regression |
| Aygun | 2022 | Turkey | cohort study | 875 | 200/675 | 49.2 | logistic regression |
| Bergenfelz | 2016 | Sweden | cohort study | 5252 | 1050/4202 | 49 | logistic regression |
| Chen | 2017 | China | cohort study | 3236 | 1044/2192 | 45.8 | logistic regression |
| Dralle | 2004 | Germany | cohort study | 29998 | 6833/22973 | 54 | logistic regression |
| Enomoto | 2014 | Japan | cohort study | 447 | 68/375 | 43.5 | logistic regression |
| Erbil | 2007 | Turkey | case-control | 3250 | 2872/378 | 47 | logistic regression |
| Godballe | 2014 | Denmark | cohort study | 6859 | 3419/3440 | 52 | logistic regression |
| Gunn | 2020 | USA | cohort study | 11370 | 2476/8894 | 53 | logistic regression |
| Han | 2024 | China | cohort study | 8340 | 1817/6523 | 55 | logistic regression |
| Heikkine | 2019 | Finland | cohort study | 866 | 153/713 | 55 | logistic regression |
| Joliat | 2017 | Switzerland | cohort study | 653 | 200/453 | 49 | logistic regression |
| Landerholm | 2014 | Sweden | cohort study | 1322 | 223/1099 | 50 | logistic regression |
| Nayyar | 2020 | India | cohort study | 228 | 100/128 | 55 | logistic regression |
| Obata | 2024 | Japan | cohort study | 543 | 128/415 | 55 | logistic regression |
| Staubitz | 2020 | Germany | cohort study | 4598 | 1056/3542 | 54 | logistic regression |
| Stopenski | 2022 | USA | cohort study | 11595 | 2522/9073 | 52.5 | logistic regression |
| Tabriz | 2024 | Germany | cohort study | 1147 | 293/854 | 51 | logistic regression |
| Thomusch | 2000 | Germany | cohort study | 7266 | 2266/5000 | 51.8 | logistic regression |
| Wolff | 2025 | Poland | cohort study | 185 | 40/145 | 43.2 | logistic regression |
