# Supplementary material for: Risk factors for recurrent laryngeal nerve injury following thyroid surgery: a systematic review and meta-analysis
Source: Front Surg. 2026 Jan 7;12:1731701. doi: 10.3389/fsurg.2025.1731701 (PMC12819817; doi:10.3389/fsurg.2025.1731701)
Supplement: Supplementary file 3 [file Table2.docx]

| case control | | | | | | | | | |
| --- | --- | --- | --- | --- | --- | --- | --- | --- | --- |
| Study | Is the case definition adequate? | Representativeness of the cases | Determination of control group | Definition of Controls | Comparability of cases and controls based on the design or analysis | Ascertainment of exposure | Same method of ascertainment for cases and controls | Non response | Total scores |
| Erbil2007 | * | * | * | * | ** | * | * | * | 9 |

| cohort study | | | | | | | | | |
| --- | --- | --- | --- | --- | --- | --- | --- | --- | --- |
| Study | Representativeness of the exposed group | Selection of non-exposed groups | Determination of exposure factors | Identification of outcome indicators not yet to be observed at study entry | Comparability of exposed and unexposed groups considered in design and statistical analysis | design and statistical analysis | Adequacy of the study's evaluation of the outcome | Adequacy of follow-up in exposed and unexposed groups | Total scores |
| Aspinall2019 | * | * | * | * | ** | * | * | * | 9 |
| Aygun2022 | * | * | * | * | ** | * | * | * | 9 |
| Bergenfelz2016 | * | * | / | * | ** | * | * | * | 8 |
| Chen2017 | * | * | / | * | * | * | * | * | 7 |
| Dralle2004 | * | * | * | * | ** | * | * | * | 9 |
| Enomoto2014 | * | * | * | * | ** | * | * | * | 9 |
| Godballe2014 | * | * | / | * | ** | * | * | * | 8 |
| Gunn2020 | * | * | / | * | * | * | * | * | 7 |
| Han2024 | * | * | * | * | ** | * | * | * | 9 |
| Heikkine2019 | * | * | * | * | ** | * | * | * | 9 |
| Joliat2017 | * | * | / | * | ** | * | * | * | 8 |
| Landerholm2014 | * | * | / | * | * | * | * | * | 7 |
| Nayyar2020 | * | * | * | * | ** | * | * | * | 9 |
| Obata2024 | * | * | * | * | ** | * | * | * | 9 |
| Staubitz2020 | * | * | * | * | ** | * | * | * | 9 |
| Stopenski2022 | * | * | / | * | ** | * | * | * | 8 |
| Tabriz2024 | * | * | / | * | * | * | * | * | 7 |
| Thomusch2000 | * | * | * | * | ** | * | * | * | 9 |
| Wolff2025 | * | * | / | * | ** | * | * | * | 8 |
